# Genome-scale flux balance analysis reveals redox trade-offs in the metabolism of the thermoacidophile *Methylacidiphilum fumariolicum* under auto-, hetero-and methanotrophic conditions

**DOI:** 10.3389/fsysb.2024.1291612

**Published:** 2024-01-29

**Authors:** Alexis Saldivar, Patricia Ruiz-Ruiz, Sergio Revah, Cristal Zuñiga

**Affiliations:** ^1^ Departamento de Procesos y Tecnología, Universidad Autónoma Metropolitana-Cuajimalpa, Ciudad de Mexico, DF, Mexico; ^2^ Department of Biology, San Diego State University, San Diego, CA, United States

**Keywords:** verrucomicrobia, thermoacidophile, methanotroph, genome-scale metabolic model, flux balance analysis

## Abstract

Members of the genus *Methylacidiphilum* are thermoacidophile methanotrophs with optimal growth temperatures between 50°C and 60°C, and pH between 1.0 and 3.0. These microorganisms, as well as other extremophile bacteria, offer an attractive platform for environmental and industrial biotechnology because of their robust operating conditions and capacity to grow using low-cost substrates. In this study, we isolated *Methylacidiphilum fumariolicum* str. Pic from a crater lake located in the state of Chiapas, Mexico. We sequenced the genome and built a genome-scale metabolic model. The manually curated model contains 667 metabolites, 729 reactions, and 473 genes. Predicted flux distributions using flux balance analysis identified changes in redox trade-offs under methanotrophic and autotrophic conditions (H_2_+CO_2_). This was also predicted under heterotrophic conditions (acetone, isopropanol, and propane). Model validation was performed by testing the capacity of the strains to grow using four substrates: CH_4_, acetone, isopropanol, and LP-Gas. The results suggest that the metabolism of *M. fumariolicum* str. Pic is limited by the regeneration of redox equivalents such as NAD(P)H and reduced cytochromes.

## 1 Introduction

Extremophile bacteria such as *Methylacidiphilum fumariolicum* are an attractive platform for industrial and environmental biotechnology. Their broad growth capabilities offer an opportunity to reduce manufacturing costs through processes without sterilization or using low-cost substrates (Ye et al., 2023). Between 2007 and 2008, a new clade of methanotrophic bacteria in the Phylum Verrucomicrobia was isolated from geothermal or volcanic environments (Dunfield et al., 2007; Pol et al., 2007; Islam et al., 2008). These strains currently belong to the genus *Methylacidiphilum* and are aerobic thermoacidophilic methanotrophs with optimal growth temperatures between 50°C and 60°C and an optimal pH between 2.0 and 3.0 (Schmitz et al., 2021). To date, three species have been identified (Hou et al., 2008; Anvar et al., 2014; Kruse et al., 2019) and three unclassified strains have been isolated (Erikstad et al., 2019; Awala et al., 2021). In addition, five complete genomes and 14 draft assemblies are available in the NCBI genome database (Hou et al., 2008; Anvar et al., 2014; Erikstad et al., 2019; Kruse et al., 2019; Awala et al., 2021).

Because of the recent discovery of the Verrucomicrobia methanotrophic clade, there is limited knowledge about their broad metabolic capabilities and their further biotechnological applications. For example, the *M. fumariolicum* str. SolV has been proven to grow heterotrophically on C2 and C3 compounds such as ethane, and propane (Picone et al., 2020), as well as autotrophically, using H_2_ as an electron source and CO_2_ as the only carbon source (Mohammadi et al., 2017). The pathway for the oxidation of propane, isopropanol, and acetone was also elucidated in a recently isolated *Methylacidiphilum* sp. IT6 (Awala et al., 2021). Moreover, it has been shown that the strain SolV can convert methanethiol (Schmitz et al., 2022) to H_2_S, and oxidize H_2_S to elemental sulfur (Schmitz et al., 2023). Their metabolic capabilities and resilience to harsh conditions make these bacteria excellent candidates for use in biofilters that treat H_2_S-contaminated gaseous streams or as biomining agents recovering Rare Earth Elements (REEs) from low-grade sources (Singer et al., 2023). Additionally, Verrucomicrobia methanotrophs can be a source of novel thermostable enzymes for the chemical and pharmaceutical industries (Gevaert et al., 2019; Schmitz et al., 2020). For example, heterologous expression of PmoD from *Methylacidiphilum* sp. IT6 enabled the construction of a whole-cell biocatalyst in the Type I methanotroph *Methylomonas* sp. DH1 used for the production of acetol from acetone (Chau et al., 2022). We expect that the range of biotechnological applications of Verrucomicrobia methanotrophs will further diversify as more strains are isolated from different environments.

Genome-scale metabolic models (M-models) can be used as a knowledge base to concentrate the available biochemical, genomic, metabolic, and physiological information of a target microorganisms (Thiele and Palsson, 2010; Monk et al., 2017). The genome functions are translated into a set of metabolic reactions encoded in a mathematical representation as a set of linear equations and constraints (Orth et al., 2010). The relationship between genotype and phenotype can be investigated from the solutions of M-models using Flux Balance Analysis (FBA) (Feist et al., 2007). Moreover, M-models enable the integration of multi-omic datasets into a single comprehensive analysis workflow (Noor et al., 2019; Arnolds et al., 2021; Passi et al., 2022). In methanotrophs, M-models have been used to study the mechanisms of electron transfer to the periplasmic methane monooxygenase (PMMO) (Lieven et al., 2018), one-carbon metabolism (Nguyen A. D. et al., 2020), metabolic adaptations to high salinity conditions (Bordel et al., 2020b), nitrate-dependent methane oxidation (Versantvoort et al., 2019), etc.

In this study, we isolated and sequenced the genome of *Methylacidiphilum fumariolicum* str. Pic. Then, we collected experimental growth phenotypes using four substrates and used this information to validate our reconstructed M-model. The model, also referred to as *i*AS473, was manually curated to comply with the most recent community standards (Laibe and Le Novère, 2007; Waltemath et al., 2011; Carey et al., 2020). This knowledgebase compiles with the latest bibliomic findings of the genus *Methylacidiphilum,* specifically the metabolism of *M. fumariolicum*. To our knowledge, this is the first manually curated genome-scale metabolic reconstruction for any methanotrophic Verrucomicrobia.

## 2 Results

### 2.1 Isolation and genome characterization

Taxonomic analysis of the raw sequencing data indicated that 96% of the sequences were classified as *Methylacidiphilum* (Supplementary Figure S1). Based on this result, a two-step assembly process was used to improve the contiguity of the recovered genome (see Methods Section 4.9). The final genome assembly had a total length of 2.4 Mb and an average GC composition of 41.31%, which are comparable to those of other genomes reported for this species by clade (Supplementary Table S2). It contains a full set of ribosomal and transfer RNA genes (3 and 47, respectively), and 469 of 471 BUSCO gene markers for Verrucomicrobia bacteria (Simão et al., 2015), including 2 fragmented and zero duplicated genes. Other assembly statistics are listed in Supplementary Table S2.

**FIGURE 1:**
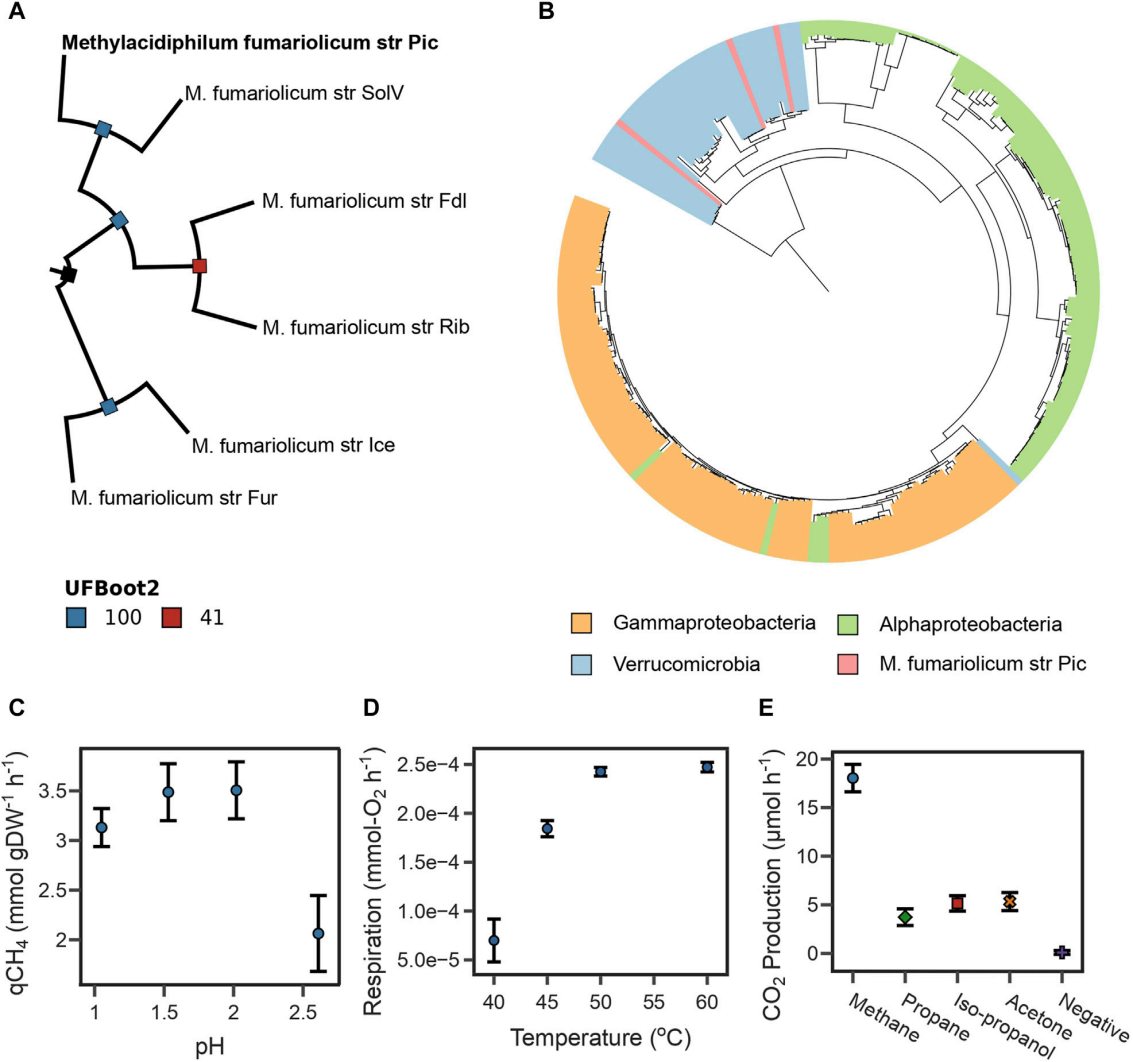
**(A)** Maximum likelihood phylogenetic tree reconstructed from the top 117 phylogenetic markers identified for *Methylacidiphilum* species. Bootstrap values were estimated using 25,000 replicates. The tree is rooted at midpoint. **(B)** Maximum likelihood phylogenetic tree for periplasmic methane monooxygenase subunit A. The sequence of strain Pic clusters with sequences of other Verrucomicrobia methanotrophs. **(C)** The highest specific CH_4_ oxidation rate from strain Pic was determined between pH 1.5 and 2.0. **(D)** The highest O_2_ respiration rate from strain Pic was determined between 50°C and 60°C. **(E)** CO_2_ production rates from strain Pic growing in four different substrates. Results show that strain Pic oxidizes C3 substrates isopropanol and acetone.

The Average Nucleotide Identity (gANI) values (Varghese et al., 2015) were calculated from orthologous gene clusters identified between this assembly and 11 genomes available for the *Methylacidiphilum* genus (see Methods Section 4.10). The genome assembly of our isolate had a gANI above 97% with all *M. fumariolicum* genomes, which exceeded the suggested cut-off of 96% for species affiliation (Sant’Anna et al., 2019). Therefore, subsequent phylogenomic analyses were conducted using five available genome assemblies for *M. fumariolicum.* The phylogenetic tree, reconstructed from the 117 top-ranking phylogenetic markers (see Methods Section 4.10), indicates that the assembly reported in this study clusters together with strain SolV in the same branch (Figure 1A). Together, the gANI values and phylogenomic analysis indicate that the recovered genome represents a novel strain of the *M. fumariolicum* species, for which the name *Methylacidiphilum fumariolicum* strain Pic is proposed, where Pic stands for the name of the municipality in which the volcanic lake is located (Pichucalco).

Furthermore, the taxonomic affiliation was predicted from the periplasmic methane monooxygenase subunit A (PmoA), which is often used as a molecular marker of methanotrophic microorganisms (Knief, 2015; Hogendoorn et al., 2021). Our genome assembly contained three complete pmoCAB operons (Supplementary Table S5). A maximum-likelihood phylogenetic tree was constructed using PmoA sequences spanning the three phyla known to have methanotrophs (Verrucomicrobia, Gammaproteobacteria, and Alphaproteobacteria). The tree indicates that all PmoA sequences from the assembly reported in this study clustered with other Verrucomicrobia methanotrophs (Figure 1B). Interestingly, most Verrucomicrobia methanotrophs encode more than one copy of the pmoCAB operon (Schmitz et al., 2021); therefore, phylogenetic analyses of a single subunit such as PmoA are inadequate for determining species-level taxonomic affiliations (Supplementary Figure S2).

### 2.2 Physiological characterization under methanotrophic and heterotrophic conditions

A key physiological characteristic of *M. fumariolicum* str. Pic is its capability to achieve high growth rates at temperatures above 50°C. Here we used the oxygen consumption rate as a response variable linked to biomass growth using a respirometry chamber. We found that the optimal growth temperatures of strain Pic were between 50°C and 60°C (Figure 1D).

We also assayed the optimal growth pH by measuring specific CH_4_ oxidation rates in experiments ranging from 1.0 to 3.0 at 50°C. As shown in Figure 1C, oxidation rates were higher between pH 1.5 and 2.0, sharply decrease after pH 2.5, and become undetectable at pH 3.0. The pH range in which strain Pic oxidizes CH_4_ is narrow in comparison to other *M. fumariolicum* strains, which can grow at pH as high as 6.0 (Pol et al., 2007). Growth rates and yields (Table 1) were determined at 50°C and pH 2.0. The CH_4_:O_2_ ratio was typical for *Methylacidiphilum* strains (1:1.6); however, the CH_4_:CO_2_ ratio of 1:0.93 was much higher than that expected for these methanotrophs (1:0.65) (Pol et al., 2007).

**TABLE 1:** Comparison of growth characteristics between *Methylacidiphilum* strains and model *i*AS473 simulations.

Strain	Substrate	Condition	qS^a^	µ (h^-1^)	Y_O2_ ^b^	Y_CO2_ ^b^	Y_X_ ^c^	Reference
Pic	CH_4_	Experimental	3.5	0.015	1.62	0.93	0.12	This Work
SolV	CH_4_	Experimental	n.d	0.070	1.6	0.65	0.35	Pol et al. (2007)
Kam1	CH_4_	Experimental	n.d	0.018	n.d	n.d	0.18	Dunfield et al. (2007)
V4	CH_4_	Experimental	n.d	0.038	n.d	n.d	0.39	Islam et al. (2008)
IT6	CH_4_	Experimental	n.d	0.047	n.d	n.d	n.d	Awala et al. (2021)
Pic	CH_4_	Simulation	3.5	0.037	1.5	0.57	0.43	This Work
Pic	CH_4_	Simulation^d^	3.5	0.029	1.6	0.66	0.34	This Work
SolV	H_2_+CO_2_	Experimental	13.2	0.047	0.32	0.19	0.19	Mohammadi et al. (2017)
Pic	H_2_+CO_2_	Simulation^e^	13.2	0.034	0.37	0.11	0.11	This Work
IT6	Isopropanol	Experimental	n.d	0.042	n.d	n.d	n.d	Awala et al. (2021)
IT6	Acetone	Experimental	n.d	0.039	n.d	n.d	n.d	Awala et al. (2021)
Pic	Propane	Simulation	1.16	0.033	3.63	1.84	1.16	This Work
Pic	Isopropanol	Simulation	1.16	0.038	2.92	1.64	1.35	This Work
Pic	Acetone	Simulation	1.16	0.033	2.63	1.84	1.16	This Work

^a^ Substrate uptake rate in units of mmol gDW^−1^ h^-1^.

^b^ Oxygen and CO_2_ yields in reference to the substrate in units of mol mol^-1^.

^c^ Biomass yields in reference to the carbon source in units C-mol mol-1, yields were calculated assuming a biomass formula weight of 24.6 C-mol gDW-1.

^d^ Simulations constraining flux of reaction FALDHpp, to be 20% of the total formaldehyde oxidation rate.

^e^ Simulations constraining flux of reaction HYD4pp to be 76% of the total H_2_ oxidation rate.

n.d., not determined.

Three pmoCAB operons (Supplementary Table S4) were identified in the Pic genome. Interestingly, the strains SolV and IT6 also have three pmoCAB operons and they have been proven to oxidize C3 substrates (e.g., IT6 can grow on isopropanol, acetone, and acetol as carbon source) (Picone et al., 2020; Awala et al., 2021). The high sequence homology between the pmoA3 of strain IT6 and strain Pic (Supplementary Table S4) provided computational evidence that strain Pic could potentially grow on C3 compounds using operon pmoCAB3 (Supplementary Figure S2). Therefore, the capacity of strain Pic to oxidize C3 compounds was evaluated by independent incubations with 50 mM acetone, 50 mM isopropanol, and 10% LP-Gas (∼90% propane and ∼10% of a mix of propylene, butylene, isobutane, and n-butane). Figure 1E shows that the CO_2_ production rates of cultures with the three substrates were higher than the negative control, but lower than cultures incubated with 10% CH_4_.

### 2.3 Genome-scale metabolic network reconstruction

#### 2.3.1 Metabolic network properties

The genome-scale metabolic reconstruction of *M. fumariolicum* str. Pic was generated using a semi-automatic methodology (see Methods Section 4.12.1). The initial draft reconstruction contained 603 genes, 1,604 reactions, and 1,555 metabolites. Out of all reactions, 492 (31.2%) had no gene association. The missing genes for these reactions were filled by manual queries (Camacho et al., 2009) against protein sequences in the KEGG pathway map for *M. infernorum* (Hou et al., 2008) or MetaCyc database (Caspi et al., 2014). Using this method, gene associations for 79 reactions were identified, while the remaining 415 reactions were removed from the model, along with 390 metabolites associated with those reactions. Furthermore, 37 stoichiometric duplicate reactions were removed, and 43 reactions that represented sub-reactions or reaction mechanisms were replaced by a lumped reaction. Of the remaining metabolites and reactions, 618 and 581 could not be annotated across databases and were removed from the model. Next, to allow the production of all biomass precursor metabolites, 101 reactions were manually gap-filled and an additional 43 were added to complete hydroxylamine oxidation metabolism, C3 substrates oxidation, autotrophic metabolism, and acid resistance mechanisms. Subsequently, reaction identifiers were translated into BiGG namespace (King et al., 2016), and 96 new reaction identifiers, associated with 79 genes, were created for non-existent reactions in this database (Supplementary Table S7).

**FIGURE 2:**
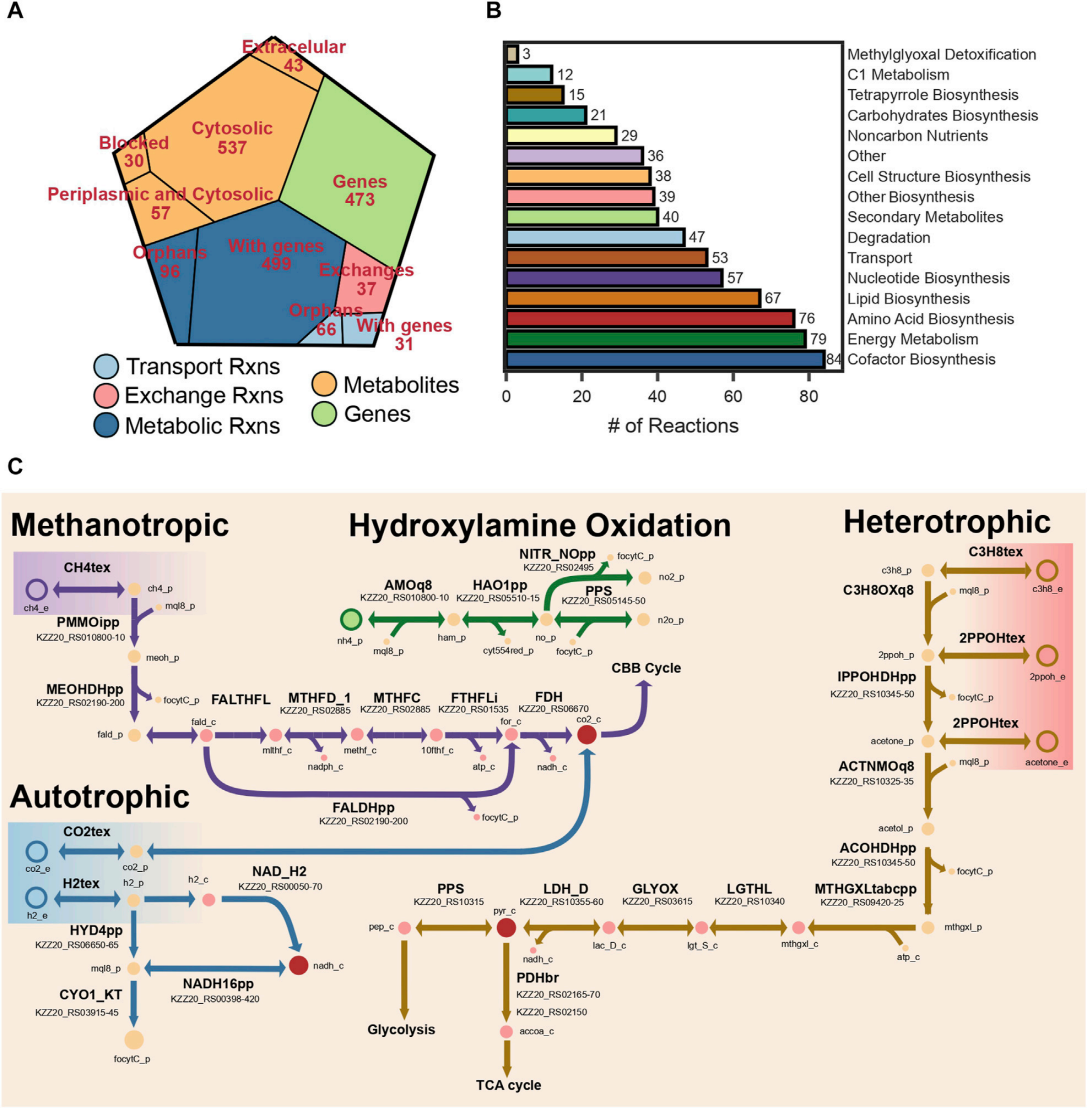
**(A)** Voronoi tree map showing the distribution of reactions, metabolites, and genes. **(B)** Bar plot showing the number of reactions grouped by pathway. **(C)** Metabolic map of the different metabolic modules represented in the model.

The final reconstruction comprised 667 metabolites, 729 reactions, and 473 genes (Figure 2A). Out of the total number of reactions 162 did not have a gene association. The reconstruction was named *i*AS473 following community standards. Standardized quality analysis with MEMOTE (Lieven et al., 2020) indicated that the model is stoichiometrically consistent, and without erroneous generation of energy metabolites (Gevorgyan et al., 2008; Lieven et al., 2020). Moreover, an annotation consistency score of 92% indicated that the model is of high quality. A detailed description of MEMOTE results may be found in the GitHub repository (see Data Availability Statement). The Model is available in SBML Level 3 version 1, with the FBC package enabled (Hucka et al., 2003; Olivier and Bergmann, 2018).

#### 2.3.2 Manual curation and biomass constraints

##### 2.3.2.1 Electron transport chain

The electron transport chain (ETC.) and energy conservation mechanisms are active in bacteria using quinones. These molecules are lipophilic compounds of the cytoplasmic membrane. Bacteria contain up to three types of quinones: ubiquinones, menaquinones, and demethylmenaquinones (Meganathan, 2001). Verrucomicrobia methanotrophs are known for producing menaquinone through a recently identified pathway using futalosine as an intermediate (Hiratsuka et al., 2008). Interestingly, the genome sequence of our strain does not encode for any of the genes necessary to produce ubiquinol. As a result, all reactions in *i*AS473 have been manually curated to use menaquinones as electron transporters.

All components of the, ETC, necessary for energy conservation (complex I-V) are encoded in the genome of strain Pic (Figure 2C), including the Alternative Complex III (ACIII) known to act as a cytochrome-menaquinol reductase in all Verrucomicrobia methanotrophs (Schmitz et al., 2021). Unfortunately, it is unclear whether ACIII contributes to the proton motive force (*pmf*) by translocating electrons across the membrane (Sousa et al., 2018; Sun et al., 2018). Because of the uncertainty in the stoichiometry of this complex, cytochrome-ubiquinol reductase activity was modeled by reaction CYO1_KT in which two protons are translocated across the membrane. The stoichiometry of the remaining components of the, ETC, was modeled by assuming a P/O ratio of 2.5 (Bordel et al., 2019a).

##### 2.3.2.2 Carbon metabolism

The pathway for CH_4_ assimilation begins with its oxidation to methanol by the enzyme methane monooxygenase (MMO) enzyme. Our model contains the PMMO which is present in the cell wall. Although the mechanisms of electron transfer to this enzyme are still under debate, previous modeling studies have suggested that electrons for CH_4_ oxidation originate from the quinone pool (Bordel et al., 2019a). In our model, menaquinones were used as electron donors in the PMMOipp reaction (Figure 2C). Gene protein reaction rule (GPR) for this reaction was set to operons pmoCAB1 and pmoCAB2 because those have the highest sequence similarity to those expressed in the presence of CH_4_ from strain IT6 (Supplementary Table S4).

Subsequently, methanol is oxidized to formaldehyde by a methanol dehydrogenase (MDH). We found that our strain encodes the lanthanide-dependent MDH XoxF, together with the periplasmic substrate-binding protein XoxJ and the cytochrome C XoxG (Supplementary Table S5), as well as the gene cluster pqqBCDE and pqqA required to produce the cofactor pyrroloquinoline used by periplasmic dehydrogenases, comprising a total of seven genes. Protein homology and experimental evidence for strain SolV showed that the cytochrome CGJ can donate electrons to a secondary cytochrome, suggesting electron transfer to a terminal oxidase (Versantvoort et al., 2019). We included those details in *i*AS473.

Methanotrophic Verrucomicrobia have been shown to exclusively use CO_2_ as a carbon source via the Calvin-Benson-Basham (CBB) cycle (Khadem et al., 2011). Because of this, the pathways for formaldehyde oxidation become highly relevant to provide electron equivalents and most of the CO_2_ used in the CBB cycle. Formaldehyde oxidation to formate proceeds via pathways involving methylene derivates of the cofactor tetrahydrofolate (THF), or the archaea-like cofactor tetrahydromethanopterin (THMP) (Chistoserdova et al., 2009). In methanotrophic Verrucomicrobia, formaldehyde could bind spontaneously or enzymatically to THF to form methylene-THF (Vorholt et al., 2000; Chistoserdova et al., 2009; He et al., 2020), and be converted to formyl-THF by the bifunctional dehydrogenase/cyclohydrolase FolD (Schmitz et al., 2021). Subsequently, formyl-THF could be converted to formate by a formate-THF-ligase accompanied by the production of ATP (Marx et al., 2003). Alternatively, formaldehyde could be oxidized directly to formate by the MDH-XoxF (Pol et al., 2014). Finally, a cytosolic formate dehydrogenase could oxidize formate to CO_2_ using NADH as an electron acceptor (Figure 2C). Genomic evidence for our strain showed that all the enzymes necessary to operate the CBB cycle and regeneration of glyoxylate (e.g., phosphoglycolate phosphatase, glycolate oxidase) are present in strain Pic (Supplementary Table S5).

Additionally, we included all reactions necessary to enable C3 metabolism in our model. We found previous genomic and transcriptomic evidence of this functions in *Methylacidiphilum* sp. IT6 while growing on propane, isopropanol, and acetone (Awala et al., 2021). In this pathway (Figure 2C; Supplementary Table S4), propane could be oxidized to isopropanol by a PMMO; however, transcriptome analyses could not resolve whether this reaction is catalyzed by PMMO3 or PMMO1 (Picone et al., 2020; Awala et al., 2021). Then, isopropanol could be converted to acetone by a glucose-methanol-choline (GMC) oxidoreductase, and acetone oxidized to acetol by PMMO3. Operon pmoCAB3 contains the gene pmoD, which was recently shown to be necessary for the oxidation of acetone (Chau et al., 2022). Finally, acetol could be converted to methylglyoxal by the same GMC oxidoreductase, and methylglyoxal assimilated into pyruvate via a three-step pathway. In the model, all reactions between propane oxidation and methylglyoxal production take place in the periplasm (Figure 2C) and use menaquinones as electron transporters (Takahashi et al., 2015). Those reactions are associated with 10 genes total in our model.

##### 2.3.2.3 Autotrophic metabolism

To date, two *Methylacidiphilum* strains (SolV and RTK17.1) have been reported to grow autotrophically using H_2_ and CO_2_ under microaerobic conditions (O_2_ saturation <2%) (Carere et al., 2017; Mohammadi et al., 2017). Our genomic evidence shows that our strain contains three hydrogenase operons, as well as the gene cluster hypBFCDE/hypA, which encodes chaperone proteins necessary for the assembly of hydrogenases (Supplementary Table S5).

The three hydrogenases belong to Groups 1d, 1h and Group 3b (see Methods Section 4.9). Group 1d hydrogenases are uptake hydrogenases that use a b-type cytochrome to transfer electrons to the respiratory chain via the quinone pool (Mohammadi et al., 2017). Group 1h hydrogenases are high-affinity membrane-bound uptake enzymes (Schmitz et al., 2020), for which the electron transfer pathway has not been elucidated yet. Finally, Group 3b hydrogenases are cytosolic enzymes which catalyze the reversible oxidation of H_2_ coupled to the reduction of NADH. We added reactions HYD4pp and NAD_H2 to the model, which represent periplasmic and cytosolic hydrogenases, respectively (Figure 2C). It is important to note that microorganisms growing on substrates with a higher redox potential than NAD(P)H produce electron equivalents via energy-driven reverse electron flow (Aleem et al., 1963; Ingledew, 1982; Poughon et al., 2001; Sapra et al., 2003; Ferguson and Ingledew, 2008). Considering this, the reaction NADH16pp (complex I) was set to be reversible (Häger and Bothe, 1987) in simulations under autotrophic conditions. Onward, we will refer to this as the reverse electron flow hypothesis.

##### 2.3.2.4 Biomass reaction

The composition of the biomass reaction was imported from the model of the gram negative methanotroph *Methylomicrobium buryatense* 5G (B1) (de la Torre et al., 2015) into the first draft of our model. This reaction was updated for *M. fumariolicum* Pic by adding experimental measurements of amino acids (see Methods Section 4.12.1). Additionally, coefficients of the biomass precursors were rescaled so that the biomass had a molecular weight of 1 g mmol^-1^ (Chan et al., 2017). The growth-associated ATP maintenance consumption (GAM) was calculated from experimental CH_4_:O_2_ ratios, and a coefficient of 10.86 mmol ATP gDW^–1^ h^-1^ was added to the biomass reaction. Supplementary Table S9 provides a detailed breakdown of biomass components.

Before gap-filling, the production of 13 biomass precursors was blocked. After extensive manual curation we added and connected reactions to produce all these components. However, we could not identify the genomic evidence necessary to produce L-homocysteine and, in consequence, L-methionine. Overall, we included the necessary orphan reactions for the two L-homocysteine production pathways described in bacteria (Belfaiza et al., 1998; Vermeij and Kertesz, 1999; Hwang et al., 2002)

### 2.4 Model validation and applications of flux balance analysis

Our model was validated by comparing predicted growth rates and growth stoichiometries with bibliomic and our experimental data for four carbon sources (CH_4_, propane, isopropanol, and acetone). Under all conditions, NH_4_ was used as the nitrogen source. Overall, model predictions were within the same order of magnitude as that of the bibliomic data (Table 1).

#### 2.4.1 Calculation of redox trade-offs in methanotrophic metabolism

To validate the model, we performed a sensitivity analysis of the growth rate while varying Growth Associated Maintenance (GAM) and Non-GAM while using CH_4_ as only carbon source. The sensitivity was calculated as the slope of the curve of growth rate vs. *GAM/NGAM* and has units of Δµ ΔGAM^-1^ or Δµ ΔNGAM^-1^. Supplementary Figure S3A shows that the model is largely insensitive to changes in the GAM, showing constant growth predictions for GAM values below 32 mmol ATP gDW^–1^ h^-1^. However, the slope changed to 1.2 × 10^−4^ for values between 32 and 100 mmol ATP gDW^–1^ h^-1^. In contrast, changes in NGAM had a substantially larger effect on the predicted growth rates, decreasing from 0.036 to less than 0.001 h^-1^ (Supplementary Figure S3B). Although the growth rate is constant below NGAM values of 4.2, from that value onward it decays with a slope of 4.5 × 10^−3^, becoming infeasible for all NGAM values above 12 mmol ATP gDW^–1^ h^-1^. The value of NGAM used for all subsequent simulations was 3.5, which was obtained from a previous model (Bordel et al., 2019b).

Additionally, we evaluated the possible effects of formaldehyde oxidation by the XoxF-MDH (reaction FALDHpp). Since this enzyme uses cytochrome C as the electron acceptor, the direct oxidation of formaldehyde to formate by XoxF-MDH prevents the production of NAD(P)H and ATP in the THF-dependent pathway (Figure 2C). Therefore, simulations showed an increased flux through this reaction. We found that it reduces the growth rate by limiting the NAD(P)H available for the CBB cycle and anabolic reactions. (Supplementary Figure S4A). Using O_2_ yields as constraint, we determined that the model showed the highest agreement with the bibliomic data when 20% of the total formaldehyde flux was oxidized in reaction FALDHpp (Table 1). Therefore, this ratio was used as a constraint in all the subsequent simulations using CH_4_.

**FIGURE 3:**
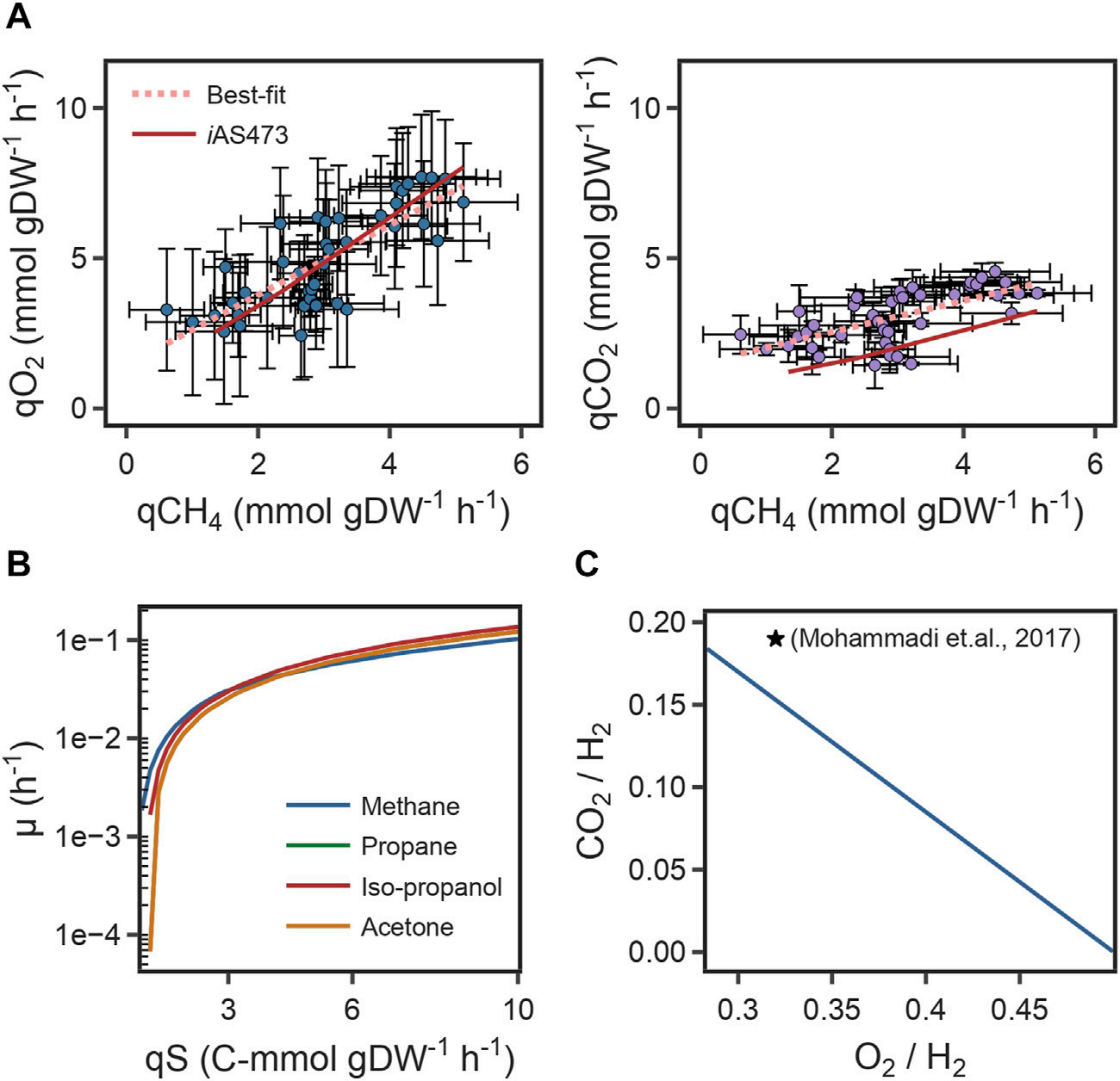
**(A)** Scatter plot of specific O_2_ uptake rates (left), CO_2_ production rates (right) as a function of specific CH_4_ consumption rates and its comparison to model predictions. **(B)** Growth rates with four different substrates as a function of carbon uptake rate. **(C)** Comparison of the predicted O_2_ and CO_2_ yields to bibliomic data under autotrophic conditions. Yields are referenced to 1 mol of H_2_.

Finally, the predicted correlation between O_2_ uptake rates/CO_2_ production rates, and CH_4_ uptake rates was compared with the experimental growth data from strain Pic (Figures 3A, B). For both components, the slope of the model was in good agreement with the slope of the line of best-fit of the experimental data (Table 2). This indicates that the model can accurately predict metabolic changes under varying environmental conditions. However, for CO_2_, the intercepts of the model and the fit were different (Table 2) because of a remarkable higher yield of CO_2_ in our strain. Those results suggest that the difference in the intercepts is caused by physiological differences in strain Pic.

**TABLE 2:** Comparison between growth phenotypic data from strain Pic and model simulations.

	Oxygen		Carbon Dioxide	
	Line of Best-Fit^a^	*i*AS473^b^	Line of Best-Fit^a^	*i*AS473^b^
Slope	1.16	1.46	0.52	0.54
Intercept	1.45	0.47	1.50	0.40
Log-Likelihood	−59.78	−62.10	−43.58	−68.53
R-squared	0.622	0.292	0.416	0.549

^a^ Ordinary least-squares parameters for experimental data of O_2_ uptake rates/CO_2_ production rates vs. CH_4_ uptake rates.

^b^ Linear correlation between O_2_ uptake rates/CO_2_ production rates vs. CH_4_ uptake rates predicted by the model.

#### 2.4.2 Calculation of redox trade-offs in autotrophic metabolism

**FIGURE 4:**
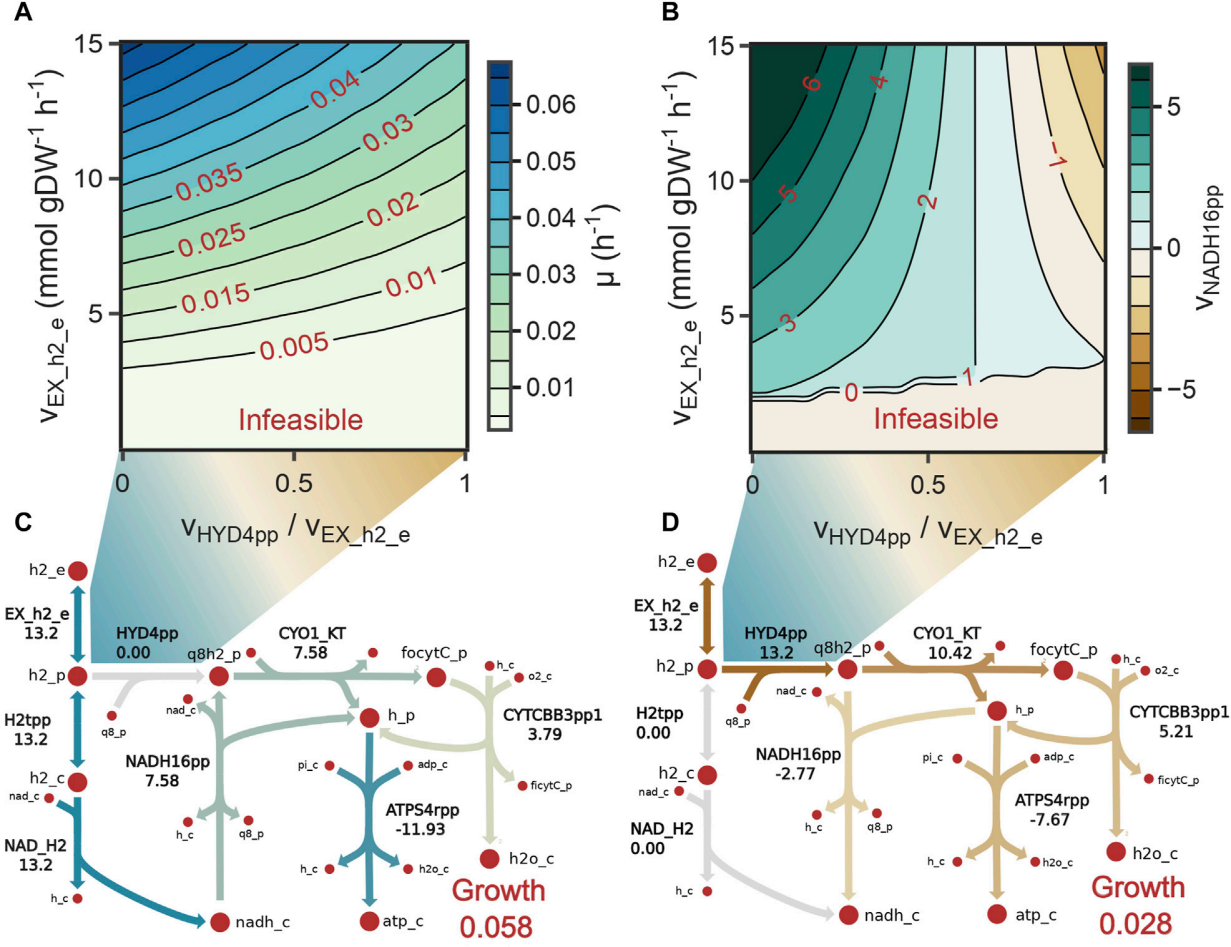
**(A)** Contour plot showing the monotonic decrease in growth rate as the fraction of H_2_ oxidized by the periplasmic hydrogenases increases (HYD4pp). **(B)** Contour plot showing the directionality of complex I (NADH16pp) as the fraction of H_2_ oxidized by HYD4pp increases. NADH16pp changes its directionality when HYD4pp oxidizes 76% of the total H_2_ flux. **(C, D)** Metabolic flux distributions of reactions in the electron transport chain when the fraction of H_2_ oxidized by HYD4pp is 0 **(C)** or 1 **(D)**. Activity of HYD4pp constraints the maximum growth rate because the *proton motive force* needs to be diverted from ATP production to NADH regeneration.

We used the model to investigate whether stoichiometric constraints support growth under the reverse electron flow hypothesis. Under this hypothesis, when H_2_ is oxidized by the periplasmic hydrogenase (HYD4pp), NADH is produced by the reverse activity of complex I in the respiratory chain (NADH16pp) at the expense of *pmf*. Phase plane analysis revealed a trade-off between this phenomenon and growth rate (Figure 4A). Similar to the results for reaction FALDHpp, as a higher fraction of H_2_ is oxidized through HYD4pp, NADH regeneration becomes a rate-limiting step in the metabolism, thereby decreasing the maximum growth rate achievable (Figures 4C, D). Additionally, *pmf* consumption reduces the achievable ATP production rate, as shown by a reduction of 55% in the flux through ATP synthase reaction (Figure 4D). Model predictions indicate that growth under the reverse electron flow hypothesis is only feasible if the total H_2_ uptake rate is higher than 3.4 mmol H_2_ gDW^–1^ h^-1^, and simulations indicated that reverse electron flow becomes necessary if approximately 76% of the H_2_ flux is oxidized through HYD4pp (Figure 4B), showing good agreement with bibliomic data (Table 1; Figure 3C).

#### 2.4.3 Heterotrophic metabolism is limited by redox reactions

Growth under heterotrophic metabolism was simulated for three different substrates: propane, isopropanol, and acetone. To make the simulations comparable between conditions, the substrate uptake rate was normalized to an equivalent carbon uptake rate of 3.5 C-mmol gDW^–1^ h^-1^, which is the carbon uptake rate measured from experiments with CH_4_. With this constraint, the predicted growth rates in C3 substrates were consistent with bibliomic data from strain IT6 (Table 1). Interestingly, the growth rate in isopropanol was remarkably higher (isopropanol = 0.038 h^-1^; propane, acetone = 0.033 h^-1^). This occurred because the conversion of isopropanol to acetone by GMC-oxidoreductases produces two extra redox equivalents in the form of protons that can potentially be supplied to the, ETC. On the other hand, when propane or acetone are used as substrates, electrons generated by GMC-oxidoreductases are consumed in the oxygenation reactions of the PMMO. The consequence is that flux of CYTCBB3pp1 (cytochrome oxidase) was 23.6% higher in isopropanol, thus enabling a higher growth rate.

**FIGURE 5:**
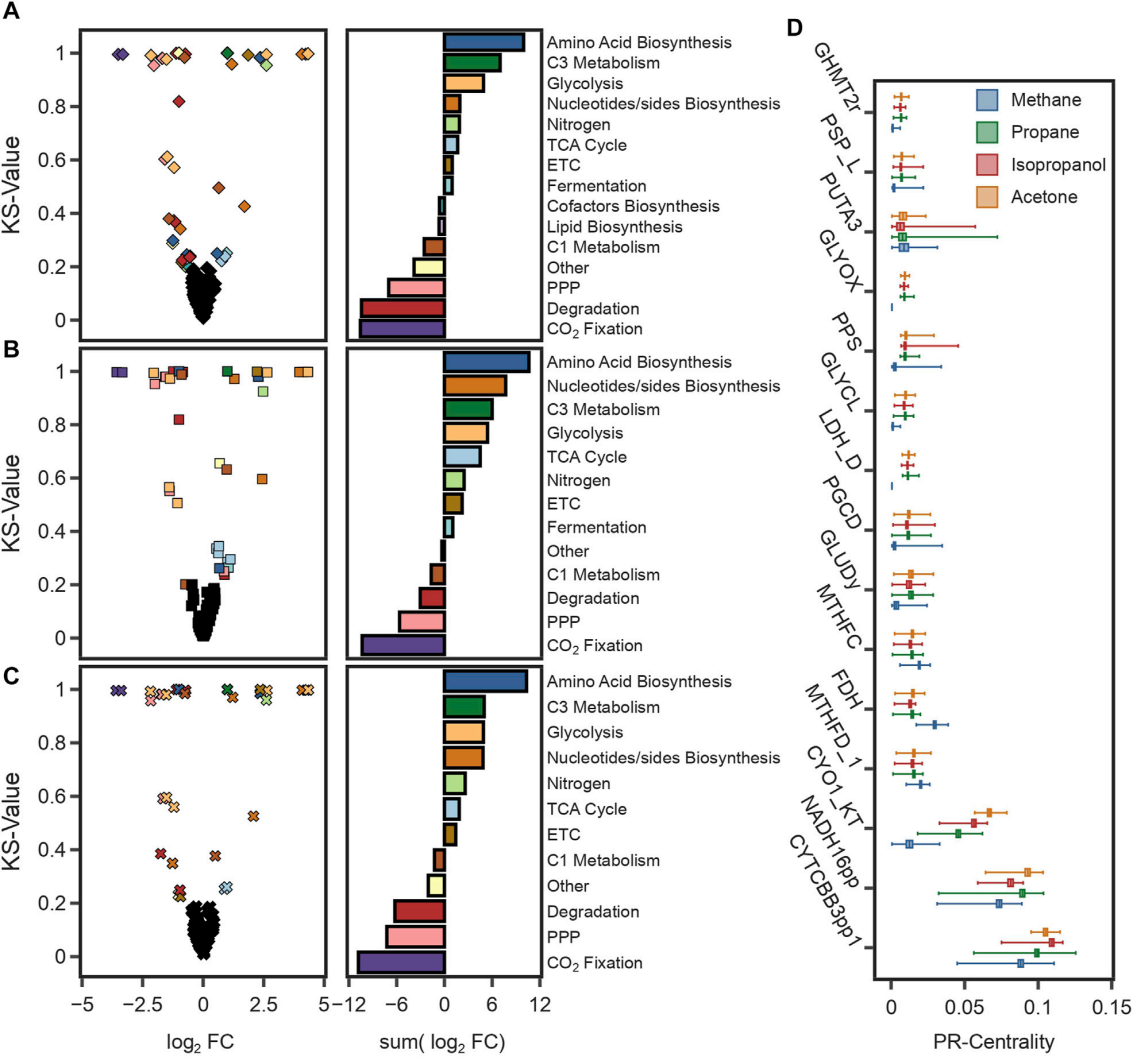
**(A–C)** The graphs on the left are volcano plots showing the median flux differences between simulations using CH_4_ and **(A)** propane, **(B)** isopropanol, and **(C)** acetone. The plot was generated with the log_2_ fold change (log_2_ FC) values from the median of 10,000 simulations and the value of the Kolmogorov-Smirnov test (KS-value). The cut-offs to identify reactions with significant differences were 0.5 for the log_2_ FC and 0.2 for the KS-value. The graphs on the right show the total flux change for reactions with significant differences grouped by pathways. **(D)** Box plot of the PageRank scores of the 17 most central reactions for 10,000 simulations in each substrate. The PageRank score is a measure of the centrality or importance of a reaction, and it is higher for reactions with a higher connectivity or reactions with a higher mass flux.

To further investigate those phenotypes, we sampled the solution space of each condition (total 4) to investigate the key differences between methanotrophic and heterotrophic metabolism. Using optGpSampler (Megchelenbrink et al., 2014), 10,000 flux distributions were simulated for CH_4_, propane, isopropanol, and acetone. Changes in predicted flux variation of reactions were identified by comparing the median fluxes using the Kolmogorov-Smirnov test static (KS-value) and the log2 fold change (log2FC) using CH_4_ as the reference condition (see Methods Section 4.13). Overall, the highest differences found were a reduction in the flux through the CCB cycle against an increase in glycolytic reactions and the TCA cycle (Figures 5A–C). Because C3 compounds are assimilated at the level of pyruvate, to produce energy and precursor metabolites carbon flux needs to be divided between the TCA cycle, and glycolytic reactions. The higher carbon content enables an increase in amino acid and nucleotides production (Figures 5A–C), with the consequential increase in growth rates (Table 1). Another key difference was the reduction in flux through the THF-dependent pathway of formaldehyde oxidation. Carbon flux through this pathway provides methylene-THF, which is used in the biosynthesis of pyrimidine deoxyribonucleosides. To compensate for its deactivation, methylene-THF was produced from glycine and serine by the glycine-cleavage-enzyme-complex (GLYCL) and the serine hydroxymethyltransferase (GHMT2r), respectively.

Furthermore, Mass Flow Graphs (MFGs) (Beguerisse-Díaz et al., 2018) were constructed for each sample to rank reactions based on their centrality, which was calculated as the PageRank value (Gleich, 2015). MFGs are weighted, directed graphs with reactions as nodes, edges that represent supplier-consumer relationships between reactions, and weights given by the mass flow between connected reactions. In all conditions, the highest-ranking reactions corresponded to those in the, ETC (Figure 5D), highlighting the energetic constraints that redox balance has on the metabolism of these microorganisms. Notably, formate dehydrogenase (FDH) was a recurring reaction in all simulations (Figure 5D). During the growth using C3 compounds, formate is a product of fermentative metabolism. Activation of fermentative reactions suggests that catabolic pathways, such as the TCA cycle, cannot meet the energy requirements on their own. Overall, these findings suggest that growth under heterotrophic conditions is limited by the production rate of redox equivalents, a result consistent with findings under methanotrophic and autotrophic conditions.

## 3 Discussion

Extremophile bacteria have the potential to lower biomanufacturing costs by reducing the energy, labor, and capital resources needed for sterilization, agitation, heating, and cooling (Levett et al., 2016; Ye et al., 2023). Moreover, extremophile bacteria are sources of novel and robust industrially relevant compounds (Tao et al., 2016) and proteins (Aulitto et al., 2017). Acidophile methanotrophs have been used for the co-degradation of organochlorine compounds (Choi et al., 2021), whereas halotolerant methanotrophs have been successfully used to produce ectoine (Cantera et al., 2017; Cho et al., 2022).

M-models have been used to study the metabolism of methanotrophs using a systems biology approach (Fu et al., 2019; Nguyen et al., 2020a), and as tools in the rational design of metabolic engineering of methanotrophs (Henard et al., 2019; Nguyen et al., 2020b). Recently, an M-model was used to study the halotolerance mechanisms of *Methylomicrobium alcaliphilum* (Bordel et al., 2020b). Although automatic reconstruction tools reduce the labor and time needed to develop M-models, extensive manual curation is still required to improve the predictive capacity (Zuñiga et al., 2020) as well as the consistency of the models with Findability, Accessibility, Interoperability, and Reusability (FAIR) principles (Wilkinson et al., 2016). In this study, we generated a high-quality, manually curated model of *M. fumariolicum* str. Pic. Although several M-models for proteobacterial methanotrophs have been published (Table 3), to our knowledge, model *i*AS473 is not only the first model available for methanotrophic Verrucomicrobia but also the first model available for any thermoacidophile methanotroph.

**TABLE 3:** List of published M-models for methanotrophic bacteria.

Name	Microorganism	Class	Reference
*i*Mb5G (B1)	*Methylomicrobium buryatense*	Gammaproteobacteria	de la Torre et al. (2015)
*i*McBath	*Methylococcus capsulatus Bath*	Gammaproteobacteria	Lieven et al. (2018)
*i*IA332	*Methylomicrobium alcaliphilum* 20 ZR	Gammaproteobacteria	Akberdin et al. (2018)
*i*MC535	*Methylococcus capsulatus Bath*	Gammaproteobacteria	Gupta et al. (2019)
No name	*Methylocysti hirsuta* CSC1	Alphaproteobacteria	Bordel et al. (2019a)
	*Methylocystis* sp. SC2	Alphaproteobacteria	
	*Methylocystis* sp. SB2	Alphaproteobacteria	
	*Methylocystis parvus* OBBP	Alphaproteobacteria	Bordel et al. (2019b)
	*Methylocella silvestris*	Alphaproteobacteria	Bordel et al. (2020a)
*i*MsOB3b	*Methylosinus trichosporium* OB3b	Alphaproteobacteria	Naizabekov and Lee (2020)

Model *i*AS473 contains 473 out of 647 that were predicted to be related to metabolic reactions in the genome assembly of strain Pic and had a MEMOTE consistency score of 92% (see Supplementary Material S1). In addition, model *i*AS473 can simulate all the known phenotypic capabilities of the *Methylacidiphilum* genus, specifically methanotrophic, autotrophic, and heterotrophic. Interestingly, under methanotrophic conditions, oxidation of formaldehyde by the XoxF-MDH prevents the production of NAD(P)H via the THF-dependent pathway. Theoretically, this should exert a negative effect on the metabolism, as the NAD(P)H pool needs to be divided between quinol regeneration, the CBB cycle, and anabolism (Keltjens et al., 2014). Indeed, the model predicts a monotonic decrease in the growth rate as a higher fraction of formaldehyde is oxidized by the XoxF-MDH. However, stoichiometric constraints on NAD(P)H regeneration could be alleviated by alternative electron transfer mechanisms not considered in this study, such as the reverse electron transfer of complexes I and III (Keltjens et al., 2014) or direct electron transfer from cytochrome C to the PMMO (Lieven et al., 2018). Although the formaldehyde oxidation activity of XoxF-MDH has only been detected *in vitro* (Pol et al., 2014), a similar functional redundancy has been observed between the THF and THMP-dependent pathways (Marx et al., 2005). It is tempting to speculate that XoxF-MDH could play a similar role in alleviating formaldehyde toxicity under transient conditions.

Model *i*AS473 predicts a similar phenomenon under autotrophic conditions. *In vitro* activity assays have shown that H_2_ oxidation in *Methylacidiphilum* species can mostly be attributed to O_2_ resistant periplasmic hydrogenases (HYD4pp) (Carere et al., 2017; Schmitz et al., 2020). However, the activity of these enzymes prevents NADH production by the O_2_ sensitive cytoplasmic hydrogenases. Although NADH could be produced by Group 3b hydrogenases (Hedderich and Forzi, 2005), these enzymes are highly O_2_ sensitive; therefore, it is not clear if their activity alone is sufficient to supply all electron equivalents required for growth in *Methylacidiphilum* species.

Simulations under autotrophic conditions showed that an increase in the fraction of H_2_ oxidized by HYD4pp decreases the growth rate because of the reduction in NADH production (Figure 4A). To compensate for this loss, complex I carries a reversible reaction to produce NADH; however, this activity decreases the available *pmf* used for ATP production, constraining the growth rate dramatically. Simulations predicted that a reverse electron flow is necessary if at least 76% of the H_2_ flux is oxidized through HYD4pp (Figure 4B), this result is consistent with activity assays between the membrane and soluble fractions of H_2_ oxidizing cells from strain SolV, in which approximately 62% of the H_2_ was oxidized by the membrane fraction (Carere et al., 2017; Schmitz et al., 2020). Since reverse electron flow is a highly endergonic process, the metabolism needs to overcome an energy threshold to make growth feasible (Poughon et al., 2001). Interestingly, model simulations predicted a threshold at 3.4 mmol of H_2_ gDW^-1^ h^-1^; This result needs to be tested experimentally and further validated using advanced modeling methodologies such as metabolism and gene expression models (Tibocha-Bonilla et al., 2022).

The changes in flux patterns between methanotrophic and heterotrophic conditions, as predicted by the model, were consistent with transcriptome analyses of strain IT6 grown in isopropanol. Model simulations indicated that under heterotrophic conditions, carbon assimilation bifurcates in pyruvate: a fraction of the carbon flux is diverted to the TCA cycle for the regeneration of the NAD(P)H pool, while the rest is diverted to glycolysis and the Pentose Phosphate Pathway to produce precursor metabolites. As expected, a significant proportion of the carbon flux was also diverted to formate and later to CO_2_ through the formate dehydrogenase reaction (FDH), suggesting that this reaction was also necessary to replenish the NAD(P)H pool key for methanotrophic metabolism. In a study by Awala et al. (2021) the authors determined that genes for phosphoenol pyruvate synthase, as well as the three components of the pyruvate dehydrogenase complex, were upregulated in isopropanol-growing cells. Moreover, 11 out of the 32 upregulated genes belonged to enzymes of the TCA cycle.

Overall, the model *i*AS473 enables a systematic process to compile available biochemical and genetic information, detect possible errors during the annotation process of the genome assembly, and identify knowledge gaps in the metabolism of *Methylacidiphilum* species. We expect that this model will be a useful tool for researchers to investigate the metabolism of this novel genus.

## 4 Materials and methods

### 4.1 Sample collection

In March 2019, we took sediment and water samples of approximately 250 mL samples from the crater-lake in “El Chichonal”, an active volcano located in the state of Chiapas in Mexico (17^o^21′N, W93 ^o^ 41′W; 1100 masl.). After the most recent eruption started in March 1982 three small lakes were created in the crater; by November 1982, one lake occupying an area of 14 ha remained (Armienta et al., 2008). Temperatures in the lake vary between 20°C and 95°C, and the pH varies between 2 and 4. The crater lake has been the source of extremophile bacteria (Ovando-Chacon et al., 2020; Ortiz-Cortés et al., 2021; Ovando-Ovando et al., 2023), and recently proteobacterial methanotrophs were identified in the sediments (Rincón-Molina et al., 2019; Rincón-Molina et al., 2020). Supplementary Table S1 contains the coordinates of the different sites. Sediment samples were collected in sterile plastic containers, and water samples were collected in sterile amber bottles. Immediately after collection, the samples were stored in ice and transported to our laboratory in Mexico City for further genomic studies.

### 4.2 Culture conditions

Cultures of sediments were incubated in gastight serum bottles of 125 mL, at a temperature of 50°C, agitation speed of 160rpm using Ammonium Mineral Salts (AMS) medium at pH 2 with, with 10% (v/v) of CH_4_ in the headspace unless otherwise specified. The medium composition is reported in Supplementary Table S3.

### 4.3 Enrichment and isolation

Approximately 1.3 g of sediments from each site were mixed and diluted with 10 mL of AMS and 10 mL of water sampled from the lake. This mixture was incubated in 125 mL of gastight serum bottles at a temperature of 40°C and an agitation speed of 200 rpm. The concentration of gases in the headspace of the bottle was adjusted to 20% (v/v) of CH_4_ and 1% (v/v) of CO_2_ by removing air with a syringe and adding the corresponding volume of each gas. This mixture was incubated until all CH_4_ in the headspace was depleted. After this, the mixture was used as the inoculum of five 1:10 serial dilutions in 20 mL of AMS. The dilutions were incubated under the same conditions described before, with the only difference being that CO_2_ was not added to the headspace. For isolation, 2 ml of the lowest dilution with growth were taken to start three rounds of 10^–11^ extinction culturing dilutions. After the third round, 2 ml of the lowest dilution with growth were transferred to 23 mL of fresh AMS media and incubated for 1 week before DNA extraction.

### 4.4 DNA extraction and sequencing

DNA was extracted from 25 mL of culture broth. The sample was centrifuged and washed twice in Phosphate Buffer (0.2M, pH 7.4). Then, the Qiagen DNeasy PowerSoil DNA Isolation Kit (QIAGEN Sciences, Germantown, MD, United States) was used following the manufacturer’s instructions. The samples were submitted to Novogene Corporation Inc (Sacramento, CA, United States) for library preparation and sequencing on an Illumina NovaSeq PE150 platform.

### 4.5 Utilization of respirometry to determine temperature phenotypes

Pre-grown cultures were incubated in 300 mL of AMS in a 1L gas-tight bottle, and 120 mL of CH_4_ were added daily until an optical density of 0.5 was reached. All respirometry experiments were performed in a custom-made glass chamber (Cabello et al., 2015) using a Clark-type polarographic dissolved oxygen (DO) probe (YSI Incorporated, United States). A data acquisition module (CompactDAQmx, NI, United States) was connected to a computer for data logging every second. Before each temperature tested (40, 45, 50, 60°C), 25 mL of pre-grown bacterial cultures were incubated in gastight serum bottles for 15min with 10% CH_4_ inside a water bath pre-adjusted to the desired temperature, with an additional 15 min incubation with air alone it the headspace. Maintenance O_2_ consumption was measured by adding 3 mL of the acclimatized bacterial suspension to the glass chamber and recording DO dynamics for 10 min. Subsequently, 10 µL of a 12M methanol solution were added to the chamber and the dynamics were recorded until DO exhaustion.

### 4.6 Determination of optimal pH

Pre-grown cultures were incubated in 300 mL of AMS in a 1L gas-tight bottle, and 120 mL of CH_4_ were added daily until the culture reached an optical density of 0.5. In each pH tested (1.0, 1.5, 2.0, 2.5, and 3.0), 25 mL of pre-grown bacterial cultures were incubated in gas-tight serum bottles with an initial CH_4_ concentration of 10% in the head space. The pH of each experiment was adjusted with a solution of H_3_PO_4_ 50% (v/v). The concentrations of CH_4_, CO_2,_ and O_2_ were measured every 2 h by injecting 200uL of the headspace into a GOW-MAC gas chromatograph. All experiments were performed in triplicate. The dry biomass weight was measured at the end of the experiment. Data collected was used to fit a linear model and calculate the CH_4_ uptake rate and CO_2_ production rate using the python package statsmodels v0.14.0 (Seabold and Perktold, 2010).

### 4.7 Evaluation of substrate uptake rates and growth rates calculations

We tested growth phenotypes on acetone, isopropanol, and LP-Gas. Pre-grown cultures were incubated in 300 mL of AMS in a 1L gas-tight bottle, and 120 mL of CH_4_ were added daily until the culture reached an optical density of 0.5. We used 25 mL of pre-grown bacterial cultures with initial concentrations of 50 mM acetone, 50 mM isopropanol and 10% (v/v) LP-Gas. Each substrate was tested in triplicates. The concentrations of O_2_ and CO_2_ were monitored for 8 h using a GOW-MAC gas chromatograph, with an interval of 1 h 15 min between each sample. Data collected was used to fit a linear model and calculate the substrate uptake rate using the python package statsmodels v0.14.0 (Seabold and Perktold, 2010). Data collected was used to fit a linear model and calculate the CO_2_ production rate using the python package statsmodels v0.14.0 (Seabold and Perktold, 2010).

### 4.8 Analytical methods used to create model constraints

CH_4_, CO_2,_ and O_2_ were measured in a GOW-MAC gas chromatograph using a CTR1 column (Alltech, United States). Helium was used as carrier gas at a flow rate of 100 mL min^-1^. The column, detector, and injector temperatures were set to 40°C, 115°C, and 50°C respectively. The detector current was set to 125 mA. Dry biomass weight was measured by vacuum filtering 25 mL of bacterial culture in pre-weighted cellulose acetate filters (pore diameter 0.2 µm, Sartorius). Filters were dried in an oven at 60°C for 24 h and then transferred to a dehumidifying chamber until constant weight.

To accurately constrain the biomass objective function of *i*AS473 we determined the amino acids profile using a Hitachi L-8900, an automated cation exchange chromatograph. This commercial amino acid analyzer automatically process biomass samples (Walker and Mills, 1995). Briefly, 4 mg of dry weight biomass samples were hydrolyzed in HCL according to a standard protocol for biological and physiological samples (Rutherfurd and Gilani, 2009). The calibration curve was done using the amino acid standard AAS 18-5 mL of sigma. This data was used as input to adjust the biomass objective function of *i*AS473 (see Supplementary Table S9).

### 4.9 Genome assembly and annotation

Illumina adapter sequences were removed from a total of 23,920,586 paired-end reads using trimommatic (Bolger et al., 2014). The quality of the adapter-free sequences was evaluated using FastQC (https://www.bioinformatics.babraham.ac.uk/projects/fastqc/). Primary genome assembly was carried out using the Spades-based (Prjibelski et al., 2020) assembler Unicycler v0.4.9 (Wick et al., 2017) with standard parameters. Subsequently, raw reads were normalized to an average coverage of 75x using BBNorm from the BBTools software suit (https://jgi.doe.gov/data-and-tools/software-tools/bbtools/). Normalized reads were mapped to the primary assembly and the mapped reads were re-assembled with Mira V5rc1 (Chevreux et al., 2004) to increase contiguity (Lui et al., 2021). Completeness of the assembly was evaluated using BUSCO V5.2.1 (Simão et al., 2015) against the subset of verrucomicrobial genes (2019–04–24). Ribosomal and tRNA presence was evaluated using Infernal cmscan v1.1.4 (Nawrocki and Eddy, 2013) against the Rfam database (Kalvari et al., 2021). The final assembly was scaffolded using SSPACE V2.0 (Boetzer et al., 2011), and Pilon (Walker et al., 2014) was used for gap filling of the scaffolds. Assembly statistics were calculated using QUAST v5.0.2 (Gurevich et al., 2013). Bowtie2 and samtools were used for alignment and sorting functions during all steps (Langmead and Salzberg, 2012; Danecek et al., 2021). The assembly was annotated using the online NCBI Prokaryotic Genome Annotation Pipeline v2021-07-01 (Tatusova et al., 2016). Hydrogenases were classified using HydDB (Søndergaard et al., 2016).

### 4.10 Genome-scale phylogenetic analysis

Genome assemblies available in NCBI for the *Methylacidiphilum* were evaluated for completeness with CheckM v1.2.2 (Parks et al., 2015). GET_HOMOLOGUES (Contreras-Moreira and Vinuesa, 2013) was used to identify orthologous gene clusters between the genome reported here and eleven genomes with a completeness higher than 90%. Gen clusters were used to calculate average nucleotide identity (gANI) values to define genus and species-level affiliation (Varghese et al., 2015; Sant’Anna et al., 2019). Our assembly had a gANI value above 96% for every *M. fumariolicum* genome. Therefore, only five genomes for *M. fumariolicum* were used for subsequent analyses. Orthologous gene clusters were classified into core and pan-genes. The core gene clusters were used as input to GET_PHYLOMARKERS (Vinuesa et al., 2018) to estimate a phylogenetic tree. The run_get_phylomarkers_pipeline shell script was used on core protein sequences with default parameters to identify proteins with optimal characteristics for phylogenetic analysis. This script outputs concatenated alignments of the optimal phylogenetic markers, which were used as input to IQ-TREE v2.2.0.3 (Minh et al., 2020) for tree estimation under the maximum likelihood criteria using UFBoot2 (Hoang et al., 2018) with 25,000 bootstrap replicates. Unrooted trees were estimated using automatic model selection with ModelFinder (Kalyaanamoorthy et al., 2017) and rooted artificially at the midpoint and they are shown in Figure 1A.

### 4.11 Phylogenetic tree reconstruction of PmoA

For PmoA, reference sequence WP_009059718.1 was used as a query for three BlastP (Camacho et al., 2009) searches against NCBI non-redundant database (Sayers et al., 2022) using taxonomic filters set to Verrucomicrobia, Alphaproteobacteria, and Gammaproteobacteria. The top 100 hits to each search were aligned using COBALT (Papadopoulos and Agarwala, 2007) with standard parameters. Partial sequences were removed from the alignments before using them as input to IQ-TREE v2.2.0.3 (Minh et al., 2020) for tree estimation under the maximum likelihood criteria using UFBoot2 (Hoang et al., 2018) with 25,000 bootstrap replicates. Unrooted trees were estimated using automatic model selection with ModelFinder (Kalyaanamoorthy et al., 2017) and rooted artificially at midpoint. A similar methodology was used to estimate the phylogenetic tree presented in Supplementary Figure S2, with the difference that the BlastP searches were limited to sequences of other Verrucomicrobia bacteria. Sequences from the *Methylacidimicrobium* genus were used as outgroup.

### 4.12 Metabolic reconstruction

#### 4.12.1 Draft reconstruction

The metabolic reconstruction was generated using our semi-automatic methodology (Tec-Campos et al., 2023). Initially, a draft-reconstruction was generated by using GenBank files (GCF_019429645.1) as input to PathoLogic in Pathwaytools v25.0 (Karp et al., 2019) and MetaCyc v25.0 (Caspi et al., 2014). Additionally, we used the model of gram negative methanotroph *Methylomicrobium buryatense* 5G (B1) as a reference (de la Torre et al., 2015). Pathologic was run with standard parameters and disabling taxonomic pruning. Subsequently, the draft was exported to an xml file and imported into Cobrapy (Ebrahim et al., 2013) for manual curation.

#### 4.12.2 Manual gap-filling

Production of each of the precursor metabolites was tested individually. For those metabolites which could not be produced, reactions were gap filled manually based on supporting information available in Metacyc and KEGG databases. To assign gene associations to reactions without one, protein sequences reported in the *M. infernorum* pathway map (Hou et al., 2008) from KEGG (Kanehisa and Goto, 2000; Kanehisa et al., 2023) were used as queries in a BLASTp (Camacho et al., 2009) search to the genome assembly reported in this study. For reactions not found in KEGG, protein sequences available in MetaCyc (Caspi et al., 2014) were used as the query. Reactions that still lacked gene associations after this step were removed from the model. Reactions needed to produce all biomass precursors were manually gap-filled following the same methodology.

#### 4.12.3 Model standardization

Annotation cross-references were taken from MetaCyc database and transformed as necessary to be compliant with the identifiers.org compact identifiers. Where possible, missing annotations were complemented using annotations from *i*ML1515 (Monk et al., 2017). Missing information after this step was manually added to the model. To ensure that the reconstruction meets community standards with the minimum information required in the annotation of models (MIRIAM)-compliant cross references (Laibe and Le Novère, 2007), metabolites and reactions that could not be annotated at least in one database other than MetaCyc were removed from the model. Finally, metabolite and reaction identifiers were translated into BiGG namespace (King et al., 2016). Metabolite formulas were taken from MetaCyc database. Where possible, missing formulas were complemented using information from *i*ML1515. Missing metabolite formulas after this step were added manually. If metabolite protonation and charges were available in the databases, these were set to a reference pH of 7.3 for the cytosol compartment, and pH of 2.0 for the periplasm and extracellular compartments. Else, mol files were downloaded from CHEBI (Degtyarenko et al., 2008) or KEGG (Kanehisa and Goto, 2000), and protonation states were predicted using ChemAxon (https://www.chemaxon.com) online Protonation Calculator. Stoichiometry of transport and periplasmic reactions were modified according to the protonation state of each metabolite. Ultimately, the MEMOTE Suite (Lieven et al., 2020) was used for quality analysis of the curated metabolic reconstruction. MEMOTE evaluates the annotation consistency across databases and standards and outputs an annotation score ranging from 0% to 100%.

#### 4.12.4 Stoichiometric balanced cycles for accurate redox estimation

To reduce the possibility of stoichiometrically balanced cycles, we assigned reactions reversibility constraints based on the following methods. First, the equilibrator-API (Noor et al., 2013; Beber et al., 2022) was used to calculate the standard Gibbs potentials of reactions. Gibbs potentials were used to assign directionality constraints if the absolute value of the reaction potential was greater than 1 kJ mol^-1^ and if the standard deviation was less than 3% of the absolute value. After this, stoichiometric balanced cycles, and erroneous energy generating cycles for 11 energy metabolites were detected and removed using a custom implementation of Algorithm 1 presented in (Gevorgyan et al., 2008). Reversibility constraints for reactions were modified based on information available in the databases.

#### 4.12.5 Biomass objective function

The composition of the biomass reaction was reconstructed from previous published models for Gram-negative methanotrophs (de la Torre et al., 2015; Akberdin et al., 2018; Lieven et al., 2018). The lipid composition was modified based on measurements from *Methylacidiphilum* species (Op den Camp et al., 2009), whereas the amino acid composition was modified from measurements from *M. fumariolicum* Pic. Furthermore, the reaction was normalized to a biomass molecular weight of 1 mmol g^-1^ (Lachance et al., 2019). The growth associated maintenance was calculated from experimental CH_4_:O_2_ ratios assuming a P/O ratio of 2.5. The constraints for non-growth associated maintenance were imported from the model of *Methylocystis hirsuta* CSC1 (Bordel et al., 2019b).

### 4.13 Model simulations

All simulations were performed in COBRApy (Ebrahim et al., 2013) using Flux Balance Analysis (Orth et al., 2010), with Optlang (Jensen et al., 2017) as an interface to CPLEX 20.1 (Cplex, 2009). CPLEX was used with automatic method selection and numerical tolerance set to 1 × 10^−9^. The python package statsmodels v0.14.0 (Seabold and Perktold, 2010) was used to calculate correlation parameters between O_2_ uptake rates/CO_2_ production rates and CH_4_ uptake rates.

Flux sampling was performed using the uniform sampler optGpSampler (Megchelenbrink et al., 2014) with standard parameters and 10,000 replicates. The model was sampled independently in 4 conditions: CH_4_, propane, isopropanol, and acetone. Differential fluxes in each condition were identified by comparing the median values using the Kolmogorov-Smirnov test static and the log2 fold change, with CH_4_ as the reference condition. The cut-offs used were 0.2 and 0.5 for the KS-value and the log2 FC, respectively. For each of the 10,000 replicates a Mass Flow Graph (MFG) was constructed using a custom implementation of the methods presented in (Beguerisse-Díaz et al., 2018). MFGs were used to rank reactions according to PageRank Centrality (Gleich, 2015). PageRank Centrality values were calculated using the python package NetworkX (Hagberg et al., 2008). Code used to run simulations and data analysis is available as Jupyter-notebooks (Rule et al., 2019) in the GitHub repository https://github.com/cristalzucsd/Methylacidiphilum_fumariolicum (see Data Availability Statement).

## Data Availability

The datasets presented in this study can be found in online repositories. The names of the repository/repositories and accession number(s) can be found in the article/Supplementary Material.
