# Supplementary material for: Genome-scale flux balance analysis reveals redox trade-offs in the metabolism of the thermoacidophile Methylacidiphilum fumariolicum under auto-, hetero-and methanotrophic conditions
Source: Front Syst Biol. 2024 Jan 29;4:1291612. doi: 10.3389/fsysb.2024.1291612 (PMC12341988; doi:10.3389/fsysb.2024.1291612)
Supplement: Supplementary file 2 [file Table1.DOCX]

Table 1. Comparison of growth characteristics between *Methylacidiphilum* strains and model *i*AS473 simulations. ^a^ Substrate uptake rate in units of mmol h^-1^ gDW^-1^. ^b^ Oxygen and CO_2_ yields in reference to the substrate in units of mol mol^-1^. ^c^ Biomass yields in reference to the carbon source in units C-mol mol^-1^, yields were calculated assuming a biomass formula weight of 24.6 C-mol gDW^-1^. ^d^ Simulations constraining flux of reaction FALDHpp to be 20% of the total formaldehyde oxidation rate. ^e^ Simulations constraining flux of reaction HYD4pp to be 76% of the total H_2_ oxidation rate. n.d., not determined.

| Strain | Substrate | Condition | qS^a^ | µ (h^-1^) | Y_O2_^b^ | Y_CO2_ ^b^ | Y_X_ ^c^ | Reference |
| --- | --- | --- | --- | --- | --- | --- | --- | --- |
| Pic | CH_4_ | Experimental | 3.5 | n.d. | 1.62 | 0.93 | 0.12 | This Work |
| SolV | CH_4_ | Experimental | n.d. | 0.070 | 1.6 | 0.65 | 0.35 | (Pol et al., 2007) |
| Kam1 | CH_4_ | Experimental | n.d. | 0.018 | n.d. | n.d. | 0.18 | (Dunfield et al., 2007) |
| V4 | CH_4_ | Experimental | n.d. | 0.038 | n.d. | n.d. | 0.39 | (Islam et al., 2008) |
| IT6 | CH_4_ | Experimental | n.d. | 0.047 | n.d. | n.d. | n.d. | (Awala et al., 2021) |
| Pic | CH_4_ | Simulation | 3.5 | 0.037 | 1.5 | 0.57 | 0.43 | This Work |
| Pic | CH_4_ | Simulation ^d^ | 3.5 | 0.029 | 1.6 | 0.66 | 0.34 | This Work |
| SolV | H_2_+CO_2_ | Experimental | 13.2 | 0.047 | 0.32 | 0.19 | 0.19 | (Mohammadi et al., 2017) |
| Pic | H_2_+CO_2_ | Simulation ^e^ | 13.2 | 0.034 | 0.37 | 0.11 | 0.11 | This Work |
| IT6 | Isopropanol | Experimental | n.d. | 0.042 | n.d. | n.d. | n.d. | (Awala et al., 2021) |
| IT6 | Acetone | Experimental | n.d. | 0.039 | n.d. | n.d. | n.d. | (Awala et al., 2021) |
| Pic | Propane | Simulation | 1.16 | 0.033 | 3.63 | 1.84 | 1.16 | This Work |
| Pic | Isopropanol | Simulation | 1.16 | 0.038 | 2.92 | 1.64 | 1.35 | This Work |
| Pic | Acetone | Simulation | 1.16 | 0.033 | 2.63 | 1.84 | 1.16 | This Work |

Table 2. Comparison between growth phenotypic data from strain Pic and model simulations. ^a^ Ordinary least-squares parameters for experimental data of O_2_ uptake rates/CO_2_ production rates vs CH_4_ uptake rates. ^b^ Linear correlation between O_2_ uptake rates/CO_2_ production rates vs CH_4_ uptake rates predicted by the model.

|  | Oxygen | | Carbon Dioxide | |
| --- | --- | --- | --- | --- |
|  | **Line of Best-Fit^a^** | ***i*AS473^b^** | **Line of Best-Fit^a^** | ***i*AS473^b^** |
| Slope | 1.16 | 1.46 | 0.52 | 0.54 |
| Intercept | 1.45 | 0.47 | 1.50 | 0.40 |
| Log-Likelihood | -59.78 | -62.10 | -43.58 | -68.53 |
| R-squared | 0.622 | 0.292 | 0.416 | 0.549 |

Table 3. List of published M-models for methanotrophic bacteria

| Name | Microorganism | Class | Reference |
| --- | --- | --- | --- |
| *i*Mb5G(B1) | *Methylomicrobium buryatense* | Gammaproteobacteria | (de la Torre et al., 2015) |
| *i*McBath | *Methylococcus capsulatus Bath* | Gammaproteobacteria | (Lieven et al., 2018) |
| *i*IA332 | *Methylomicrobium alcaliphilum* 20ZR | Gammaproteobacteria | (Akberdin et al., 2018) |
| *i*MC535 | *Methylococcus capsulatus Bath* | Gammaproteobacteria | (Gupta et al., 2019) |
| No name | *Methylocysti hirsuta* CSC1 | Alphaproteobacteria | (Bordel et al., 2019a) |
|  | *Methylocystis sp.* SC2 | Alphaproteobacteria |  |
|  | *Methylocystis sp.* SB2 | Alphaproteobacteria |  |
|  | *Methylocystis parvus* OBBP | Alphaproteobacteria | (Bordel et al., 2019b) |
|  | *Methylocella silvestris* | Alphaproteobacteria | (Bordel et al., 2020a) |
| *i*MsOB3b | *Methylosinus trichosporium* OB3b | Alphaproteobacteria | (Naizabekov and Lee, 2020) |
