# Supplementary material for: Genome-scale flux balance analysis reveals redox trade-offs in the metabolism of the thermoacidophile Methylacidiphilum fumariolicum under auto-, hetero-and methanotrophic conditions
Source: Front Syst Biol. 2024 Jan 29;4:1291612. doi: 10.3389/fsysb.2024.1291612 (PMC12341988; doi:10.3389/fsysb.2024.1291612)
Supplement: Supplementary file 3 [file Table3.DOCX]

Supplementary Material

Genome-scale flux balance analysis reveals redox trade-offs in the metabolism of the thermoacidophile Methylacidiphilum fumariolicum under auto-, hetero- and methanotrophic conditions

Alexis Saldivar, Patricia Ruiz-Ruiz, Sergio Revah, Cristal Zuñiga*

*** Correspondence:** Cristal Zuñiga: czuniga2@sdsu.edu

# Supplementary Text

## Reconstruction of Hydroxylamine Detoxification

Owing to the structural similarity between CH_4_ and NH_4_, the latter acts as a competitive inhibitor of CH_4_ oxidation by PMMO (Nyerges and Stein, 2009). When PMMO oxidizes NH_4_, the product is the toxic compound hydroxylamine, which also acts as an inhibitor of MDH (Duine and Frank, 1980). Therefore, most methanotrophs possess metabolic elements necessary for hydroxylamine detoxification. Hydroxylamine oxidoreductase (HAO) is the first of these components. This enzyme catalyzes the 3-electron oxidation of hydroxylamine to NO (Versantvoort et al., 2020). The haoABC genes in ammonia oxidizers encode a catalytic subunit, a multi-heme cytochrome c554, and a cytochrome cm552 with quinone reductase function (Kim et al., 2008). Electrons recovered from hydroxylamine oxidation are proposed to be transferred from cytochrome c554 to cm552 and then channeled into the electron transport chain via the quinone pool, in a process named as “Hydroxylamine Ubiquinone Redox Module” (Whittaker et al., 2000; Hooper et al., 2004). This process allows ammonia oxidizers to grow autotrophically by coupling NH_4_ oxidation with energy conservation (Klotz and Stein, 2008).

Strain Pic possesses a HAO-like protein; however, haoAB genes from *Methylacidiphilum* species lack the cytochrome cm552 that would enable quinone reductase activity, suggesting that these bacteria cannot connect hydroxylamine oxidation to energy conservation. However, the interaction of these cytochrome components with the quinone pool has not yet been experimentally tested. Therefore, alternative pathways connecting cytochrome c554 to terminal oxidases are still possible. Nonetheless, there is no indication of which component could act as a redox partner for cytochrome c554 in Verrucomicrobia methanotrophs; therefore, a demand reaction for cyt554 was added to enable hydroxylamine oxidation in *i*AS473 (Figure 2C).

The detoxification process continues with the conversion of NO to less toxic nitrogen oxides. There are two plausible mechanisms for this conversion. First, under aerobic conditions NO could react non-enzymatically with oxygen to produce NO_2_^-^ and NO_3_^-^ (Hughes, 2008). Indeed, NO_2_^-^ is the principal end-product in aerobic chemostat cultivations of strain SolV grown on H_2_ and NH_4_. Nevertheless, transcriptome analysis under this condition detected the overexpression of the copper-dependent nitrite reductase NirK (Mohammadi et al., 2017b). Although this enzyme, which catalyzes the reduction of NO_2_^-^ to NO, is linked to dissimilatory denitrification, several studies have proposed that it can also catalyze the opposite reaction (Wijma et al., 2004; Cho et al., 2006; Cantera and Stein, 2007; Caranto and Lancaster, 2017; Lancaster et al., 2018; Lehtovirta-Morley, 2018), suggesting that NirK could produce the NO_2_^-^ detected in experiments with strain SolV. In addition, strain Pic encodes the NADH-dependent NO_2_^-^ reductase NirBD (Figure 2C), suggesting a plausible reassimilation pathway for NO. Alternatively, under microoxic conditions, NO can be reduced to N_2_O by the enzyme NorBC found in *Methylacidiphilum* species as well as in strain Pic (Schmitz et al., 2021). Accordingly, when strain SolV was grown in an microoxic chemostat, NO_2_^-^ production ceased, while the reduction rate of NO to N_2_O increased about 100-fold (Mohammadi et al., 2017b). Although this pathway is like respiratory denitrification, Verrucomicrobia methanotrophs do not have any homologs for the nitrous oxide reductase that would allow further reduction of N_2_O to molecular nitrogen.

# Supplementary Figures and Tables

## Supplementary Figures


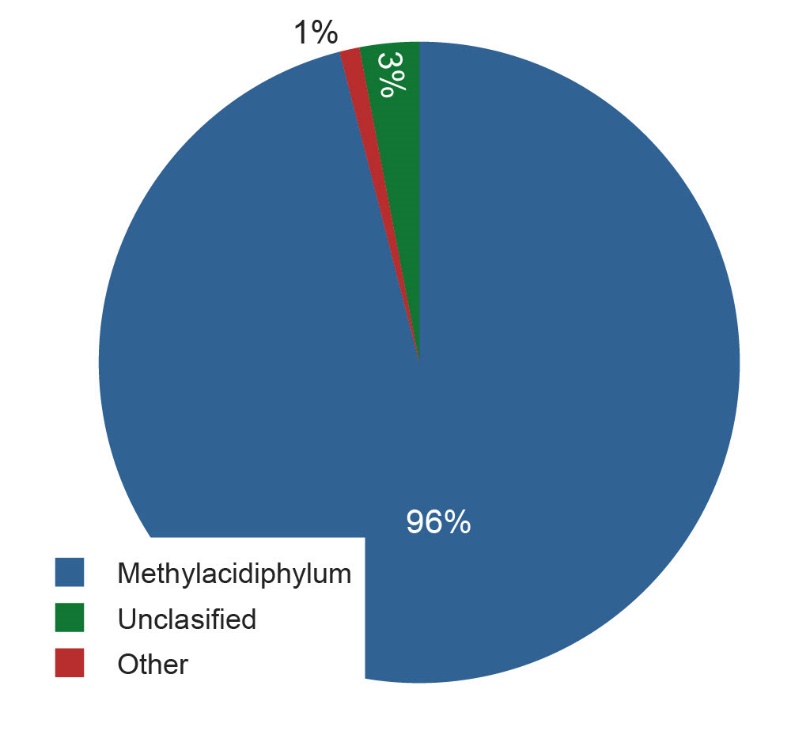


**Supplementary Figure S1.** Kaiju results


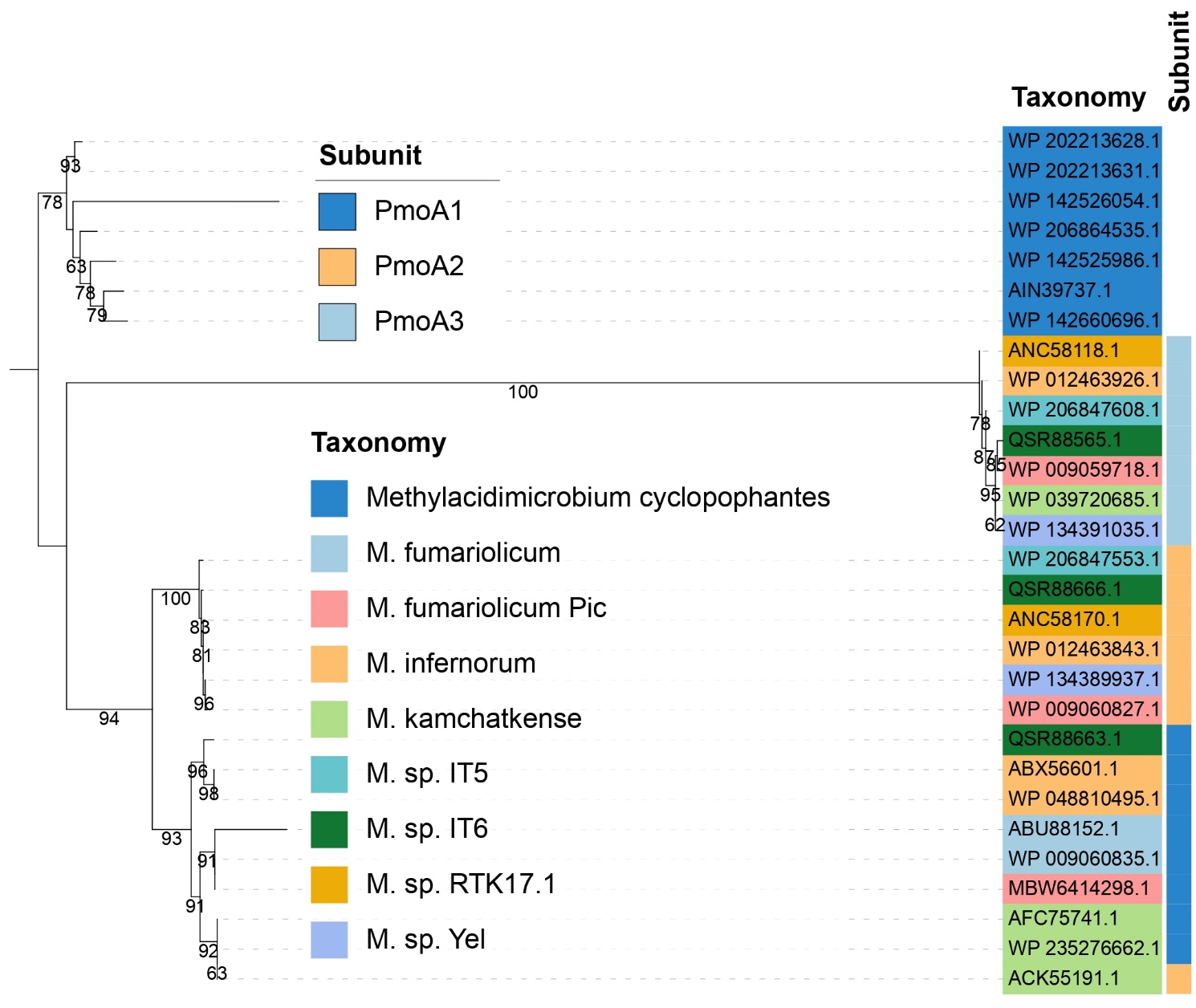


**Supplementary Figure S2.** Maximum-likelihood phylogenetic tree of periplasmic methane monooxygenase subunit A. Tree is rooted using *Methylacidimicrobium* sequences as an outgroup. The reconstruction suggests that *Methylaciphilum* pmoA subunits are clustered in three groups.


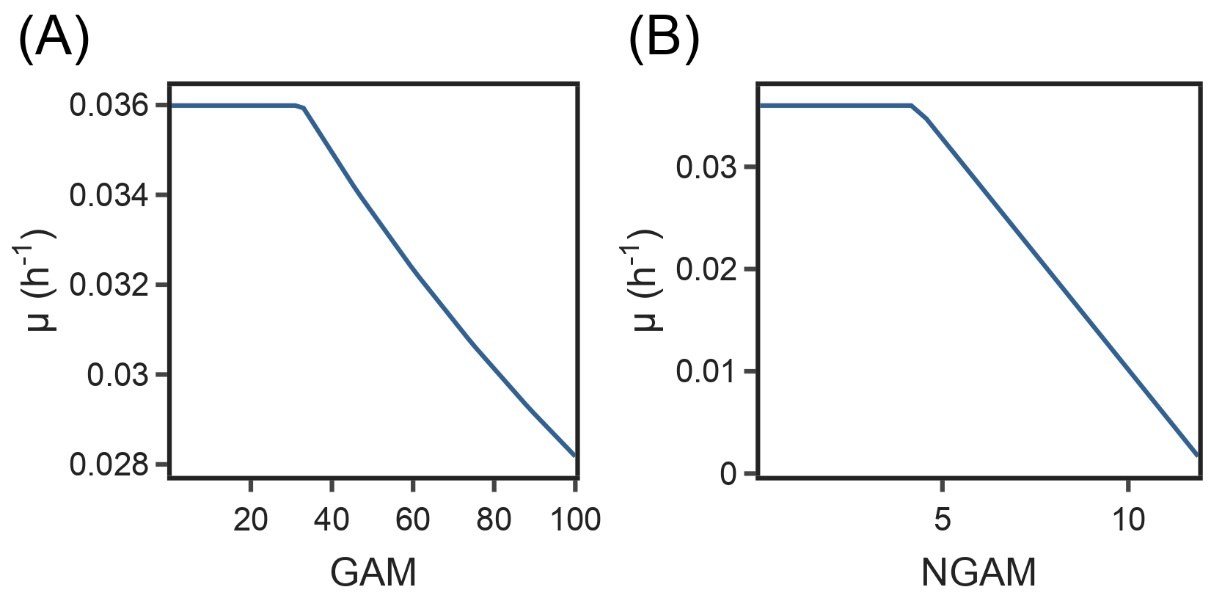


**Supplementary Figure S3.** *i*AS473 model sensitivity to changes in Growth Associated Maintenance (A) and Non-Growth Associated Maintenance (B).


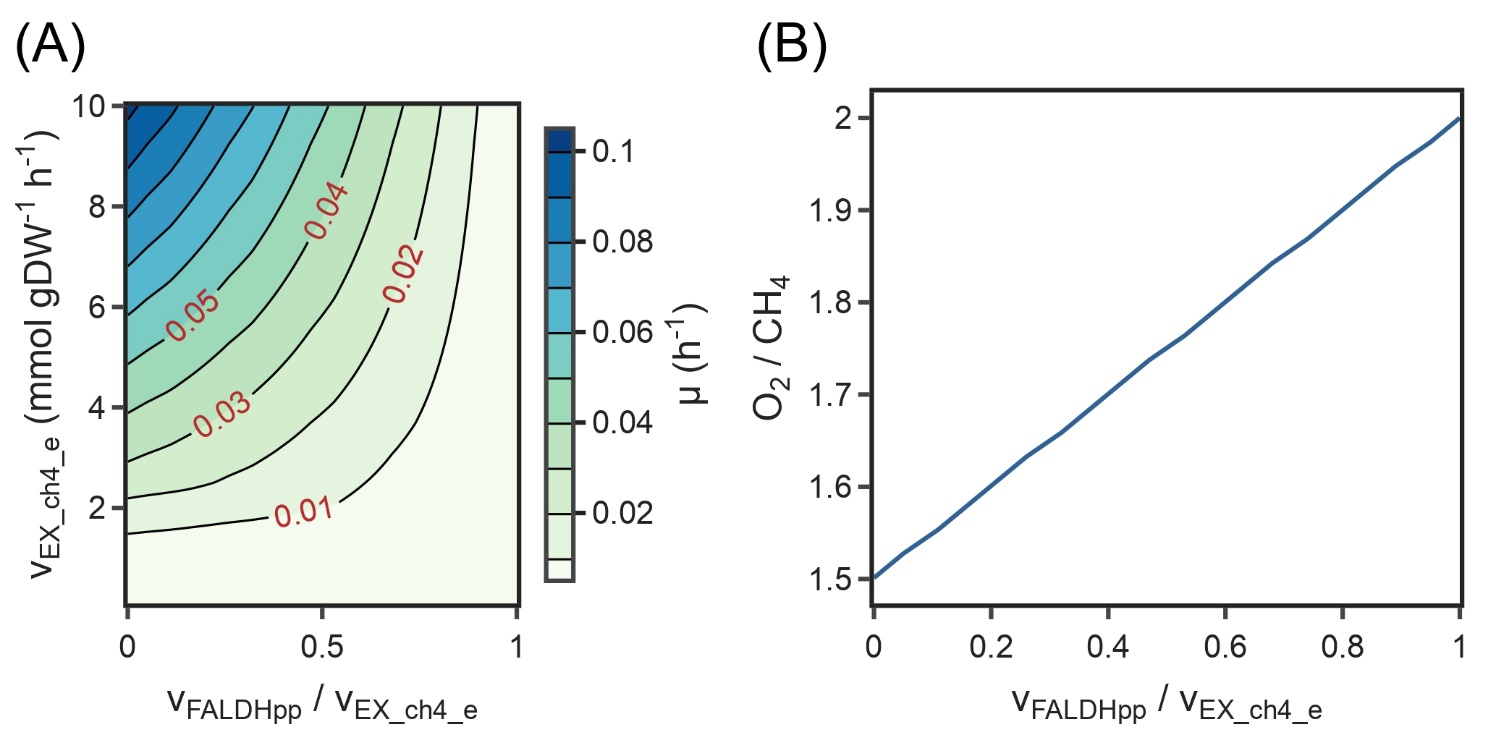


**Supplementary Figure S4.** (A) Contour plot showing the monotonic decrease in growth rate as the fraction of formaldehyde oxidized by the MDH-XoxF increases (FALDHpp). (B) Relationship between O2 yields and the fraction of formaldehyde oxidized by FALDHpp.


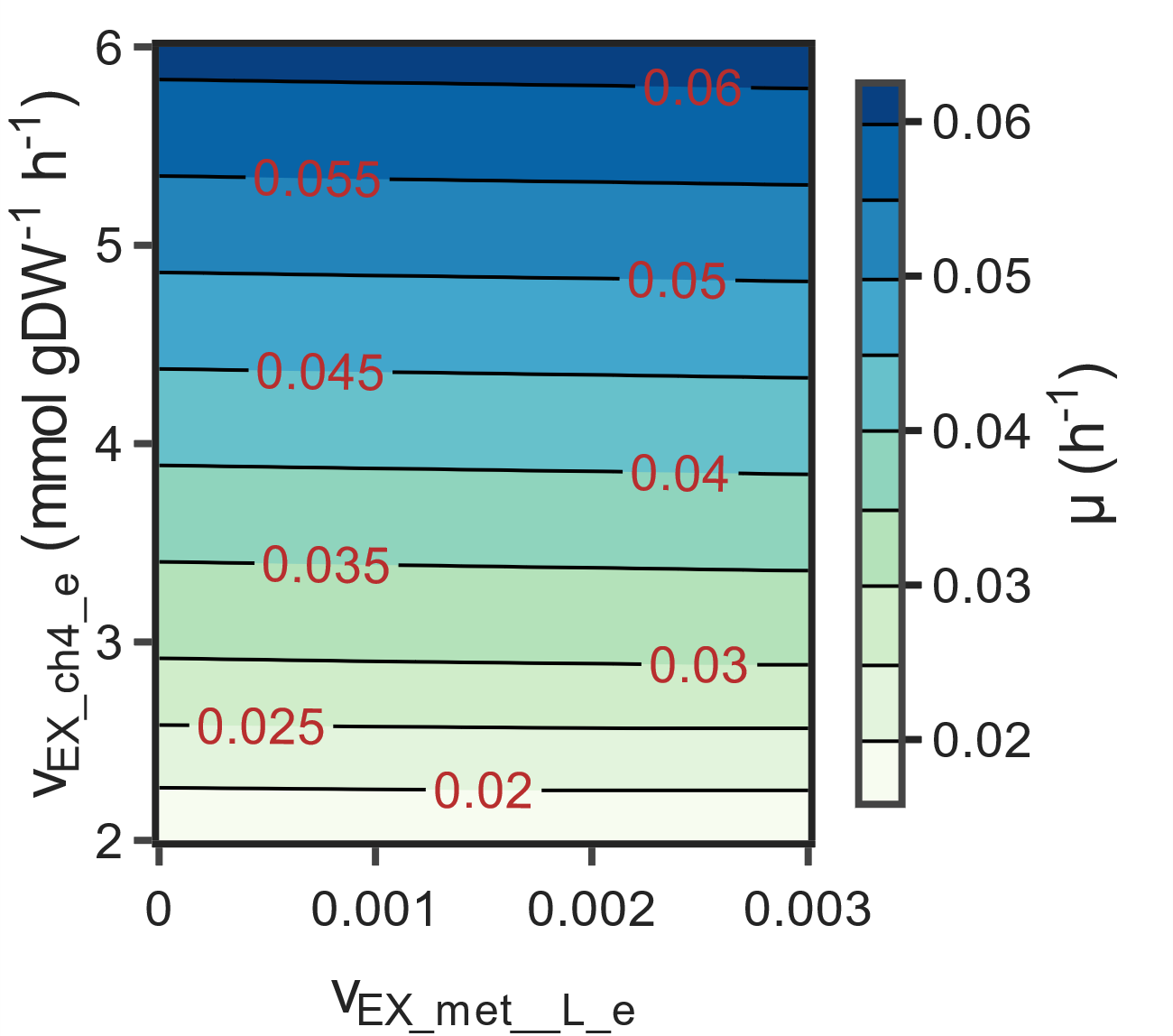


**Supplementary Figure S5.** Contour plot showing the phase plane analysis between CH_4_ oxidation and methionine transport via an ATP-dependent transport reaction. Simulations predict a decrease in growth rate of only 1.6% using the ATP-dependent transporter.

## Supplementary Tables

**Supplementary Table S1.** Coordinates of Collection Sites.

|  | Coordinates |
| --- | --- |
| Site 1 | 17°21'31.0"N 93°13'45.0"W |
| Site 2 | 17°21'35.0"N 93°13'40.1"W |
| Site 3 | 17°21'40.0"N 93°13'41.4"W |

**Supplementary Table S2.** Genome statistics for *M. fumariolicum* assemblies available in NCBI.

|  | Pic | Ice | Fur | Fdl | Rib | SolV |
| --- | --- | --- | --- | --- | --- | --- |
|  |  | GCF_004421165.1 | GCF_004421195.1 | GCF_004421175.1 | GCF_004421155.1 | GCF_949774925.1 |
| **Total Length (bp)** | 2.4Mb | 2.4Mb | 2.4Mb | 2.4Mb | 2.4Mb | 2.5Mb |
| **Number of Scaffolds** | 48 | 81 | 95 | 80 | 101 | 1 |
| **N50** | 144.9kb | 56.2kb | 74.2kb | 54.5kb | 53.9kb | 2.5Mb |
| **GC content** | 41% | 41% | 41% | 40.5% | 41% | 41% |
| **CDS** | 2,189 | 2093 | 2170 | 2149 | 2159 | 2238 |
| **Pseudo Genes** | 53 | 53 | 53 | 53 | 53 | 53 |
| **BUSCO*** | S:469, F:2, n:471 |  |  |  |  |  |
| **Coverage** | 78x | 600x | 960x | 1500x | 878x | 100x |

**Supplementary Table S3.** Composition of Ammonium Salts Mineral medium. A 50% (v/v) solution of H_3_PO_4_ was used to adjust the pH to 2.0.

|  | g L^-1^ |
| --- | --- |
| (NH_4_)_2_SO_4_ | 1 |
| MgSO_4_*7H_2_O | 0.05 |
| FeSO_4_ *7 H_2_O | 0.002 |
| NaCl | 0.02 |
| KH_2_PO_4_ | 0.12 |
| CaCl_2_*2H_2_O | 0.01 |
| LaCl3*7H2O | 0.011 |
| Trace elements Solution | 0.1% v/v |
|  |  |
| Trace Elements Solution | |
| Na_2_EDTA | 0.5 |
| CuSO_4_*5H2O | 0.22 |
| ZnSO_4_*7H2O | 0.44 |
| Na2MoO_4_*2H2O | 0.06 |
| NiCl_2_*6H2O | 0.002 |
| H_3_BO_3_ | 0.1 |
| MnSO_4_*H_2_O | 0.15 |
| CoCl_2_ | 0.18 |

**
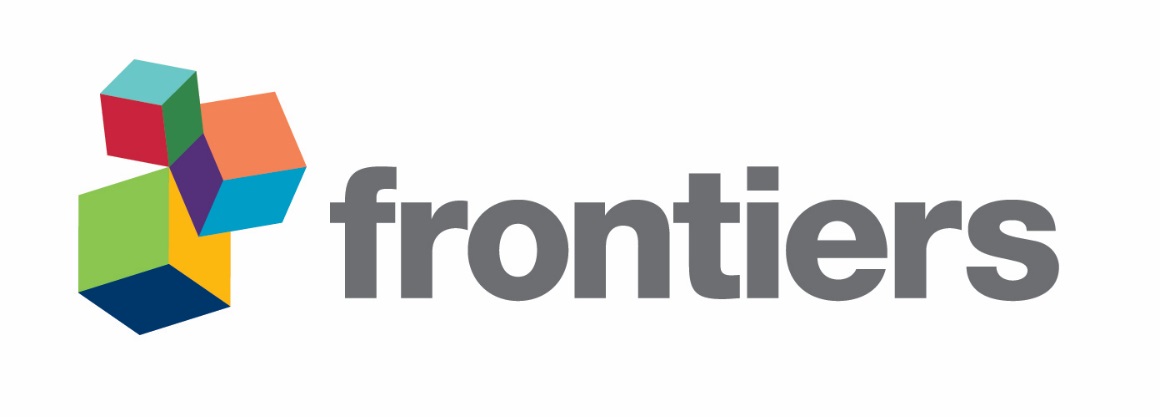
**
